# Cell wall target fragment discovery using a low‐cost, minimal fragment library

**DOI:** 10.1002/1873-3468.70281

**Published:** 2026-01-14

**Authors:** Kaizhou Yan, Mathew Stanley, Olawale Raimi, Andrew T. Ferenbach, Helge C Dorfmueller, Daan M. F. van Aalten

**Affiliations:** ^1^ Division of Molecular, Cell and Developmental Biology, School of Life Sciences University of Dundee UK; ^2^ Medical Research Council Protein Phosphorylation and Ubiquitylation Unit University of Dundee UK; ^3^ Department of Molecular Biology and Genetics Aarhus University Denmark; ^4^ Division of Molecular Microbiology, School of Life Sciences University of Dundee UK

**Keywords:** cocktail, crystallography, fragment

## Abstract

Fragment‐based inhibitor design is an established and widely used approach in drug discovery pipelines. Despite several examples of drugs originating from this approach, the identification of fragments still suffers from issues with solubility, reactivity, cost and worldwide accessibility. Here, we design a low‐cost minimal fragment library (LoCoFrag100) for crystallographic screening, with an average cLogP of 0.03 (median 0.23) and an average of £20/g for each compound, facilitating assembly in any laboratory. Formatted in a 10 × 10 matrix to minimize Tanimoto similarity in the 20 cocktails, we demonstrate its applicability on three structurally distinct enzymes involved in microbial cell wall synthesis. Hit rates range from 1 to 6% among these enzymes, with three fragments suggesting avenues for inhibitor exploration.

**Impact Statement**

LoCoFrag100 is a low‐cost, easily accessible fragment library that enables rapid survey of target ligandability in any laboratory, providing evidence to prioritise targets for follow‐up research.

## Graphical abstract

LoCoFrag100 is a fragment library made up of 100 different compounds. Similarity between the fragments is minimized and 10 different fragments are mixed into a single cocktail, which is soaked to protein crystals. These crystals are analysed by X‐ray crystallography, revealing the binding modes of the bound fragment ligands.

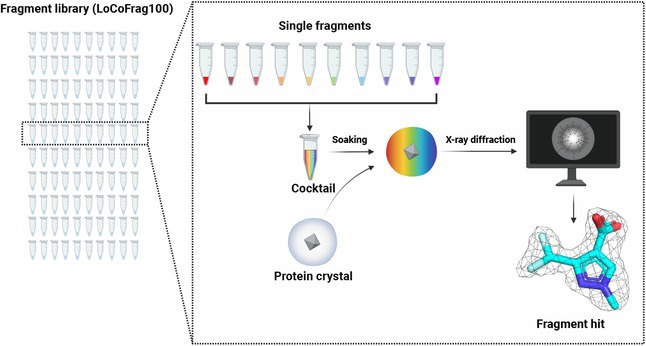

## Abbreviations

**FBLD**, fragment‐based ligand discovery

**GacA**, dTDP‐4‐dehydrorhamnose reductase

**GAS**, Group A Streptococcus

**LoCoFrag100**, low‐cost minimal fragment library with 100 compounds

**PAINS**, pan‐assay interference compounds

**PGI**, phosphoglucose isomerase

**PROTAC**, proteolysis‐targeting chimera

**SAR**, structure–activity relation

**TOI**, targets of interest

**UAP1**, UDP‐GlcNAc pyrophosphorylase

Since the mid‐1990s, fragment‐based ligand discovery (FBLD) has mainly been used to identify small‐molecule ligands [1]. The key idea of FBLD is to identify fragments (small molecules complying with the ‘rule of three’ [2]) that bind to targets of interest (TOI), often in different but proximal pockets [1, 3]. These fragments are then developed into larger high‐affinity inhibitors using the conventional structure‐based approaches [4]. By 2024, fragment‐based inhibitors have contributed to the identification of seven FDA‐approved drugs (Table 1) [5, 6, 7, 8, 9, 10, 11, 12, 13, 14, 15, 16, 17, 18, 19, 20, 21]. In cases where target inhibitors are difficult to obtain, TOIs can alternatively be degraded by proteolysis‐targeting chimeras (PROTACs)—small molecules that degrade proteins by hijacking the ubiquitination system [22, 23]. These PROTACs can also be developed by the fragment‐based approach. To date, several PROTACs are undergoing phase I/II clinical trials (e.g., Clinical Trial Numbers: NCT03888612, NCT03374085, NCT06028230), although no PROTAC has been clinically approved to treat diseases.

**Table 1:** Clinically approved drugs developed by the fragment‐based approach (by 2024).

Drugs	Types	Targets	Diseases	References
Vemurafenib	Inhibitor	Serine/threonine kinase (BRAF^V600^)	Melanomas	[5]
Asciminib	Inhibitor	Tyrosine kinase (BCR‐ABL1)	Leukaemia	[6, 7]
Venetoclax	Inhibitor	anti‐apoptotic proteins (BCL‐2)	Leukaemia	[8, 9, 10]
Erdafitinib	Inhibitor	Receptor tyrosine kinase (FGFR)	Urothelial carcinoma	[11]
Pexidartinib	Inhibitor	colony‐stimulating factor‐1 receptor	Tenosynovial giant cell tumour	[12, 13, 14]
Sotorasib	Covalent Inhibitor	GTPase (KRAS^G12C^)	Solid Tumours	[15, 16, 17, 18]
Capivasertib	Inhibitor	Protein kinase B	Breast Cancer	[19, 20, 21]

Usually, the fragment identification campaigns start with screening fragment libraries through biophysical approaches [24]. Fragment hits, even with only micromolar binding affinity, are typically identified from the screening campaign. As a prerequisite for subsequent derivation of fragments, their binding modes need to be determined through structural biology methods, with X‐ray crystallography as the most widely used approach [25]. Although the fragment‐based approach has now been widely used for decades, several limitations remain in the utilization of this approach. One significant hurdle is that although biophysical approaches can appear to result in promising fragment hits with high affinity, the binding mode of many of those cannot be determined at the atomic level (e.g., by X‐ray crystallography). An alternative strategy is to bypass the biophysical screening and directly use crystallography for the first‐round screening, that is crystallographic screening, which is usually carried out by crystal soaking [25, 26]. The binding modes of fragments could be directly revealed by crystal structures of protein–fragment complexes obtained from crystallography. Previous studies have shown that the crystallographic approach can even reveal fragment hits that are not identified by conventional biophysical approaches [26]. Despite these advantages, the direct crystallographic approach suffers from the issue of low throughput. Although platforms (e.g., XChem, FragMAX, HZB F2X) for high‐throughput crystallographic screening of compounds bound to crystallized drug targets have been established [27, 28, 29, 30], the utilization of these platforms is restricted due to limited accessibility.

Another strategy to speed up crystallographic screening is the ‘cocktailing approach’, that is mixing several fragments in one aliquot to soak a crystal [31, 32, 33]. As such, fragment libraries can be screened by fewer crystal soaking experiments, through which the screening throughput increases. Furthermore, since the cocktailing approach requires fewer crystals, this approach is suitable for proteins with limited crystal availability. However, access to commercial fragment cocktail libraries is restricted since limited numbers of vendors (for instance, Fluorine Fragment Cocktails from Life Chemicals, https://lifechemicals.com/fragment‐libraries/fluorine‐fragment‐cocktail‐library) provide fragment cocktail libraries. Although it is possible to purchase single fragments and assemble them into cocktails in‐house, the high price of some fragments hinders the assembly of cocktail libraries, especially for newly established laboratories where budget is a concern. Further limitations are that some fragments are exclusive to certain vendors with other practical issues (e.g., synthesis lead times, resupply issues and logistics costs). Furthermore, crystallographic screening usually employs high concentrations (millimolar) of fragments for crystal soaking, requiring the water solubility of such fragments to be high. To enhance the solubility, one strategy is to add a proportion of organic solvent [e.g., dimethyl sulfoxide (DMSO)] in fragments aliquots, but organic solvents may also affect the diffraction quality of protein crystals. Moreover, some commercial fragment libraries may contain pan‐assay interference compounds (PAINS) [34]. Although PAINS scaffolds may still be developed into effective ligands [35], these scaffolds are argued to be more prone to false‐positive outcomes in assays and, in turn, interfere with the fragment derivation. However, the method and experimental setup used in any screening format is an important consideration in which to judge a hit molecule for PAINS‐like characteristics.

Here, we address these issues through a low cost, accessible fragment cocktail library with 100 aqueous soluble fragments (LoCoFrag100), designed for cocktail fragment screening by crystallography. By screening LoCoFrag100, we identify fragments for three structurally distinct enzymes involved in microbial cell wall biosynthesis. Our results demonstrate that LoCoFrag100 is a broadly functional library for crystallographic screening.

## Materials and methods

### Expression and purification of proteins

*Af*UAP1 was produced as described previously [36]. After purification by size exclusion chromatography, the buffer was exchanged to 25 mm Tris–HCl pH 7.5 for crystallization.

The gene of the full‐length *Ca*PGI was codon‐optimized using the online tool available at the IDT website, *Bam*HI and *Not*I sites were added and the sequence was ordered as a geneblock. This was cloned as a *Bam*HI‐*Not*I fragment into a modified version of pGEX6P1 (GE Healthcare, Chicago, IL, USA) in which a 6‐His is added to the N terminus of the GST tag. The expression and purification were performed according to [37]. Briefly, the expression was carried out in *Escherichia coli* BL21(DE3)pPlysS by the induction of 250 μm IPTG at 18 °C for 20 h. Cells were lysed using the same method as *Af*UAP1. The protein was enriched from the supernatant by Ni beads and further purified by size exclusion chromatography (HiLoad 26/600 Superdex 75 pg, GE Healthcare) with the buffer (25 mm Hepes‐NaOH pH 7.5, 150 mm NaCl).

GacA was expressed and purified according to our previous publication [38] with modification. The gene of full‐length GacA was cloned into the 6His‐modified version of pGEX6P1 (GE Healthcare) mentioned above. The protein was expressed in *E. coli* NiCo21(DE3) by 500 μm IPTG at 18 °C for 20 h. The purification was performed by Ni beads and followed by size exclusion chromatography.

### X‐ray crystallography of proteins

*Af*UAP1 (in 25 mm Tris–HCl pH 7.5) was incubated with 2 mm MgCl_2_ and 10 mm GlcNAc‐1P on ice for at least 30 min. Crystals were formed by the sitting‐drop approach at 18 °C. The mother liquid of crystals was 0.2 M sodium acetate, 25% polyethylene glycol (PEG) 3350. Crystals were flash‐frozen in liquid nitrogen, and diffraction datasets were collected at Diamond Light Source. Data sets were processed by the Diamond processing pipelines. The phase problem was solved by molecular replacement using the reported *Af*UAP1 structure (PDB 6TN3) [39] as the search model. Refinement was carried out by refmac5 [40] or Phenix [41, 42, 43]. Protein models were built by COOT [44], and protein figures were generated by PyMOL [45].

To solve the structure of *Ca*PGI, the protein was incubated with 5 mm Glc‐6P on ice for at least 30 min. Crystals were formed in the same way as *Af*UAP1 with the mother liquid of 0.1 M MgCl_2_, 0.1 M Hepes‐NaOH pH 7.0, 21% PEG 4000. Collection of diffraction data was carried out by an in‐house diffractometer (Rigaku), and data processing was performed by iMOSFLM [46] with the structure of *Af*PGI (PDB 7OYL) as the search model for molecular replacement. To obtain *Ca*PGI crystals for the crystallographic screening, the *Ca*PGI protein was incubated with 10 mm 5‐phosphoarabinonic acid on ice for at least 30 min, and the crystallization was carried out using 0.1 M MgCl_2_, 0.1 M Hepes‐NaOH pH 7.0, 21% PEG 4000. Data were collected at Diamond Light Source, and data processing was carried out by Diamond processing pipelines with the previously obtained *Ca*PGI structure (PDB 9FZT) as the search model for molecular replacement.

The crystallization condition of GacA was the same as in [38]. Diffraction data were collected at Diamond Light Source, and data processing was also by Diamond processing pipelines with the structure of GacA from Group A *Streptococcus* (PDB 4WPG) [38] as the search model for molecular replacement.

### Crystallographic screening for the fragment cocktail library

To construct LoCoFrag100, we started from 800 000 compounds from Fluorochem. Initially, we filtered compounds with metals (heavy/transition metals, metal salts) as well as compounds from fragment libraries (Maybridge and Prestwick) we have used in the past. This filtering results in approximately 180 000 compounds, which underwent the secondary filtering by the criteria: purity at least 90%, price below £40 with the quantity over 250 mg, molecular weight between approximately 100 Da and 350 Da, XLogP between −5 and 1. The secondary filtering results in approximately 3000 compounds. With the utilization of FAF‐Drugs server [47] plus manual analysis, these 3000 compounds were subjected to the tertiary filtering to remove compounds with unwanted features including covalent modifiers, toxicity, mutagenicity, tumorgenicity, irritancy and undesirable chemical functionalities. The tertiary filtering results in approximately 1300 compounds, and these compounds were clustered in Datawarrior to select those compounds with the following features: cLogP below 1, hydrogen bond donor/acceptor number below 4. Manual selection was also applied in Datawarrior to remove problematic (e.g., putative toxic, reactive) compounds. This round of filtering resulted in approximately 800 compounds, which underwent further analysis (both automatically and manually) in Datawarrior with the consideration of chemical space coverage and Tanimoto similarity, resulting in the final selection of 200 compounds to purchase. In parallel, the original 800 000 compounds were also filtered against 30 000 protein ligands in PDB, and this filtering results in approximately 3600 compounds with protein–ligand complex structures. These 3600 compounds were subjected to another round of filtering with the following criteria: semipermissive rule of three, no covalent warheads, no undesirable functionalities and price below £40 of at least 250 mg. This round of filtering results in approximately 50 compounds. After case‐by‐case manual selection in these 50 compounds, 25 compounds were finally selected to purchase. In total, 225 compounds were purchased from Fluorochem, with the price of £4664.52 (total price of £3887.10 plus VAT (value added tax) of £777.42). On receipt of compounds, they were stored as recommended by the supplier or at < 10 °C.

Having purchased those 225 fragments, compounds that were not in a solid form were removed. The rest of those compounds (powder form) were dissolved in Milli‐Q water. For those that were unable to dissolve in Milli‐Q water, acid (1 M HCl) or base (1 M NaOH) were used as the solvent—on the assumption of no (or negligible) chemical degradation occurred. Fragment concentrations in stocks were aiming to reach 1 M, albeit actual concentrations vary due to the compound solubility. Finally, 100 fragment aliquots were obtained for the assembly of LoCoFrag100.

To make those 100 fragments into cocktails, a 10 × 10 matrix with minimal average pairwise similarity of columns/rows was made. Briefly, a 100 × 100 distance matrix of fragments was generated using a KNIME workflow based on Tanimoto similarity (‘Pubchem’ CACTVS fingerprint, Data S1) [48]. According to the 100 × 100 distance matrix, fragments were arranged into 10 × 10 matrices (Data S2) by randomly swapping compound pairs until the average pairwise similarity reaches the minimal (i.e., maximal ‘dissimilarity’) (Data S3). The final 10 × 10 matrix shows an average pairwise similarity of 0.26, with the maximal similarity of 0.70. Fragments were cocktailed by lanes and rows according to the 10 × 10 matrix. For each cocktail, 3 μL of each fragment stock was added into one Eppendorf tube, and the mixture was dried by a centrifugal vacuum concentrator. The resulting pellet was dissolved by the mother liquid of crystals. As such, the fragment cocktail has been made. Theoretically, the final concentration of each fragment is approximately 70 mm during soaking, albeit some fragments may precipitate after being mixed with other compounds.

Crystallographic screening was carried out by soaking crystals with fragment cocktails. Prior to crystal soaking, each fragment cocktail underwent a rapid pulse centrifuge to ensure that minor insoluble precipitates were removed, leaving the resulting supernatant for crystal soaking. For *Af*UAP1 and *Ca*PGI, each cocktail was equally mixed with a crystal drop under the microscope. Usually, crystals were soaked for approximately 40 min and then flash‐frozen in liquid nitrogen. GacA crystals were transferred to fresh cocktail drops and soaked for approximately 40 min, then crystals were also frozen in liquid nitrogen. The determination of crystal structures was carried out as described above.

## Results and discussion

### Design of a minimal, cost effective 100‐fragment library suitable for cocktail soaking

**Fig. 1:**
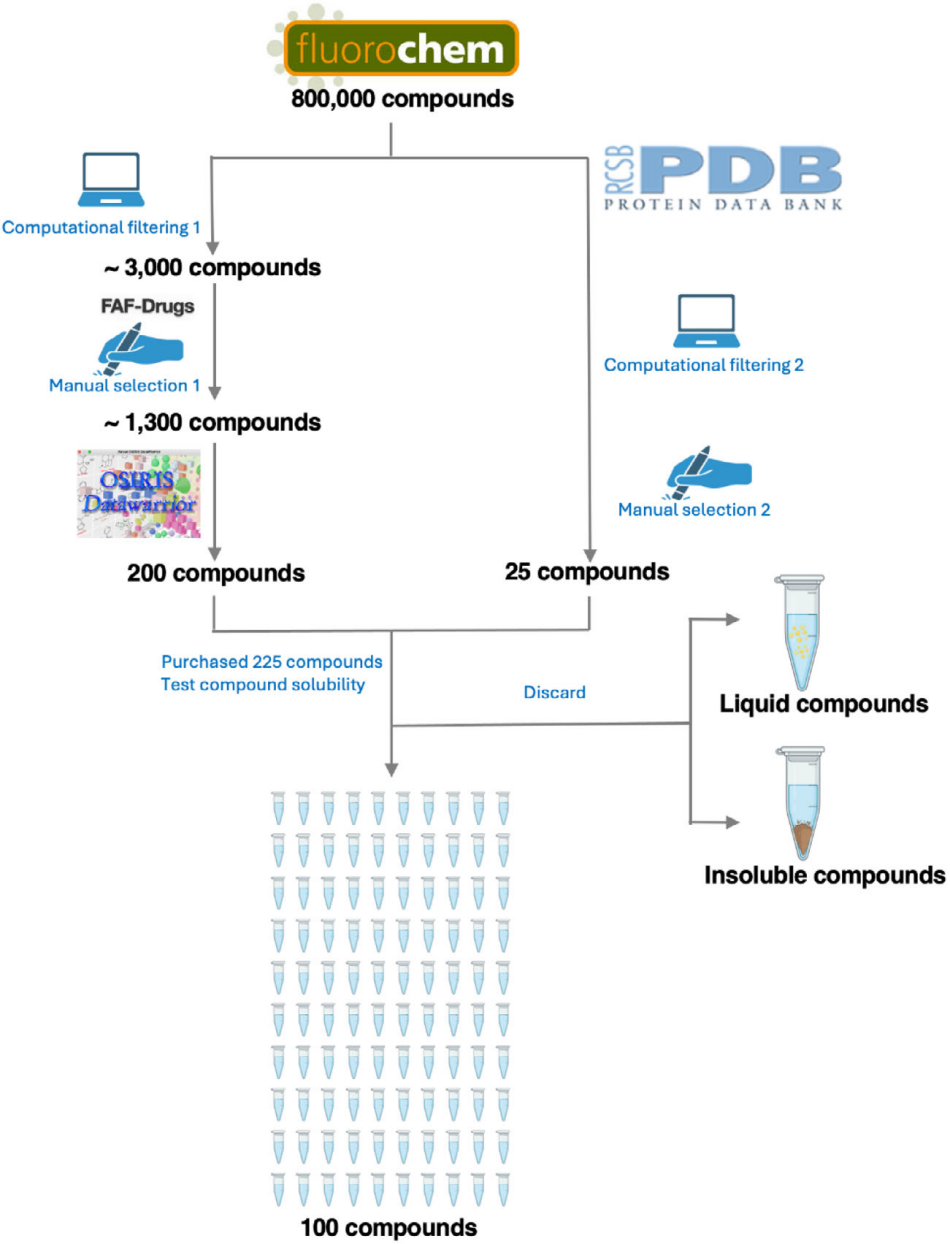
Selection of fragments for LoCoFrag100. Fragments were selected from Fluorochem by two routes. Computational filtering 1: computationally applied cutoff criteria: not in fragment libraries (Maybridge and Prestwick) used previously, no metals (heavy/transition metals, metal salts), purity at least 90%, price below £40 with the quantity of at least 250 mg, molecular weight between 100 Da and 350 Da, predicted XLogP ranges from −5 to 1, Manual selection 1: manually remove unwanted compounds (covalent modifiers, putative toxic compounds, mutagenic compounds, tumorigenic compounds, irritant compounds and compounds with less desirable functional groups) not captured by FAF‐Drugs server. Computational filtering 2: computationally applied cutoff criteria; deposited in PDB with protein‐ligand complex structures, approximate rule of three, price below £40 with the quantity of at least 250 mg. Manual selection 2: further remove other fragments with undesirable features (e.g., covalent modifiers, unwanted functionalities). The figure was drawn in BioRender.

We set out to design a low cost, accessible fragment cocktail library with 100 aqueous soluble fragments (LoCoFrag100), designed for cocktail fragment screening by crystallography. The criteria for fragment selection included approximate “rule of three” criteria (molecular weight ~ 300 Da, clogP ≲ 3, the number of hydrogen bond donors and acceptors ≲ 3 and the number of rotatable bonds ≲ 3), solubility, reactivity, affordability, chemical diversity and drug‐likeness. For each fragment, we also considered whether structures of proteins, in complex with that fragment, had been deposited in the RCSB Protein Data Bank (PDB). Using these criteria (Fig. 1), initially 225 fragments were purchased from a single supplier, Fluorochem (https://fluorochem.co.uk). Since cLogP of these 225 fragments ranges from −2.59 to 1.15, these fragments were expected to overall favour aqueous solubility. As such, aqueous solutions (Milli‐Q water, 1 M HCl or 1 M NaOH) were utilized as the solvent of each fragment, with the attempted concentration approximately 1 M (Table S2). No organic solvents were involved in these fragment aliquots. Fragments, which are insoluble or in liquid form, were not selected for the construction of library. Eventually, 100 aliquots of fragments were made. These 100 aliquots constitute LoCoFrag100 and are used for making cocktails as described in follow‐up sections.

With regard to LoCoFrag100, the average cost of fragment is approximately £20/g (in 2018). To assess the proportion of PAINS scaffolds in LoCoFrag100, the library was analysed by SwissADME [49]. Although one fragment (**27**) harbours a PAINS moiety (catechol), the remaining 99 compounds are PAINS‐free. The average cLogP value is 0.03 with the range from −2.59 to 1.15 (Tables S1 and S2).

We also analysed the ‘follow‐up’ feasibility of LoCoFrag100. We defined a hypothetical ‘exit vector’ as any heavy atom with at least one attached hydrogen that could be replaced to grow the molecule, within a pragmatic set of criteria (Table S2). In terms of hypothetical ‘exit vectors’, the library contains both ‘Ring’ (aryl and saturated) and ‘Chain’ (alkyl) vectors, with a median of 4 and 2 exit vectors per fragment molecule, respectively. In terms of vectors that support hypothetically ‘facile’ chemical elaboration based upon typically robust heteroatom coupling chemistry (e.g., ‘amide’ coupling and N‐/O‐alkylation), the library has a median of 1 Nitrogen/Oxygen exit vector per fragment molecule. Ten compounds do not formally meet the criteria for N and O exit vectors (Fragment **32**, **62**, **66**, **74**, **79**, **113**, **130**, **134**, **136, 224**). However, five of them (Fragment **32**, **62**, **66**, **74**, **79**) do contain chemical functionality amenable to further chemical elaboration. For example, halogen‐aryl coupling chemistry (fragment **62**), phenolic hydroxy group alkylation chemistry (Fragment **32**, **66**, **74**), and further amide elaboration potential (Fragment **62** and **74**). For the remaining five fragments that do not meet these criteria (Fragment **113**, **130**, **134**, **136**, **224**), substructure searching using commercial molecule aggregator services indicates the commercial availability of >> 1000 related small molecule analogues for subsequent ‘SAR‐by‐Catalogue’ activities and to inform medicinal chemistry elaboration strategies. These results indicate that all of the fragment molecules are ‘hypothetically’ capable of supporting additional chemical elaboration through available ‘exit vectors’ or have available commercial analogues to inform and support fragment elaboration strategies.

Bespoke ‘make on demand’ libraries also provide an option for custom screening libraries (for example, Enamine REAL). However, arguably ‘make‐on‐demand’ libraries are best deployed once initial crystallographic poses are available to enable rapid, vector‐focused SAR. By contrast, the minimal deck reported here is a prevalidated, physically available library for crystallographic screening and is enriched for practical exit vectors allowing elaboration once a hit is identified.

We used two commercial libraries (Jena and MiniFrag) (Table 2) [50, 51] for crystallographic fragment screening. Overall, the three libraries (LoCoFrag100, Jena and MiniFrag) are broadly complementary in property space, diversity and elaboration vectors (Fig. S1, Table S1). Across medians (Table S1), LoCoFrag100 (MW 154, cLogP 0.23, PSA 52 Å^2^) sits between the MiniFrag (MW 96, cLogP ‐0.40, PSA 45 Å^2^) and Jena (MW 180, cLogP ‐1.66, PSA 87 Å^2^) libraries, combining aromatic‐rich, mid‐weight scaffolds with balanced polarity. LoCoFrag100 shows higher ring exit‐vector counts and greater aromatic content than both comparators, likely favouring *π*‐driven binding modes (e.g., *π*‐stacking) and ring‐based expansion vectors (Table S1). Comparatively, the MiniFrag library likely provides maximal solubility, rigidity, and a higher fraction of sp3 carbons (Fsp [3]) in a lower overall molecular weight framework (Table S1). By comparison, the Jena library likely contributes greater polarity, hydrogen bonding capacity and flexibility and is therefore potentially well placed for probing polar networks and stereoselective contacts (Table S1). However, as has been seen in our own campaigns, we have failed to achieve outputs with this library. Like MiniFrag, LoCoFrag100 can be considered for initial hotspot identification activities, and preferentially for elaborating hydrophobic/*π*‐stacking interactions with clear ring‐vector geometry optionality for fragment elaboration.

**Table 2:** Comparison of LoCoFrag100 with commercial crystallographic libraries.

Libraries	Size	Main features	Cocktail or not	No. crystals needed	Hit rate (%)	References
LoCoFrag100	100	Cocktailing; Cheap; No cryoprotectant	Yes	20	1–6	This study
Jena	96	known protein ligands	No	96	9.4	[50]
MiniFrags	80	Ultra small molecules	No	80	44 (average)	[51]

**Fig. 2:**
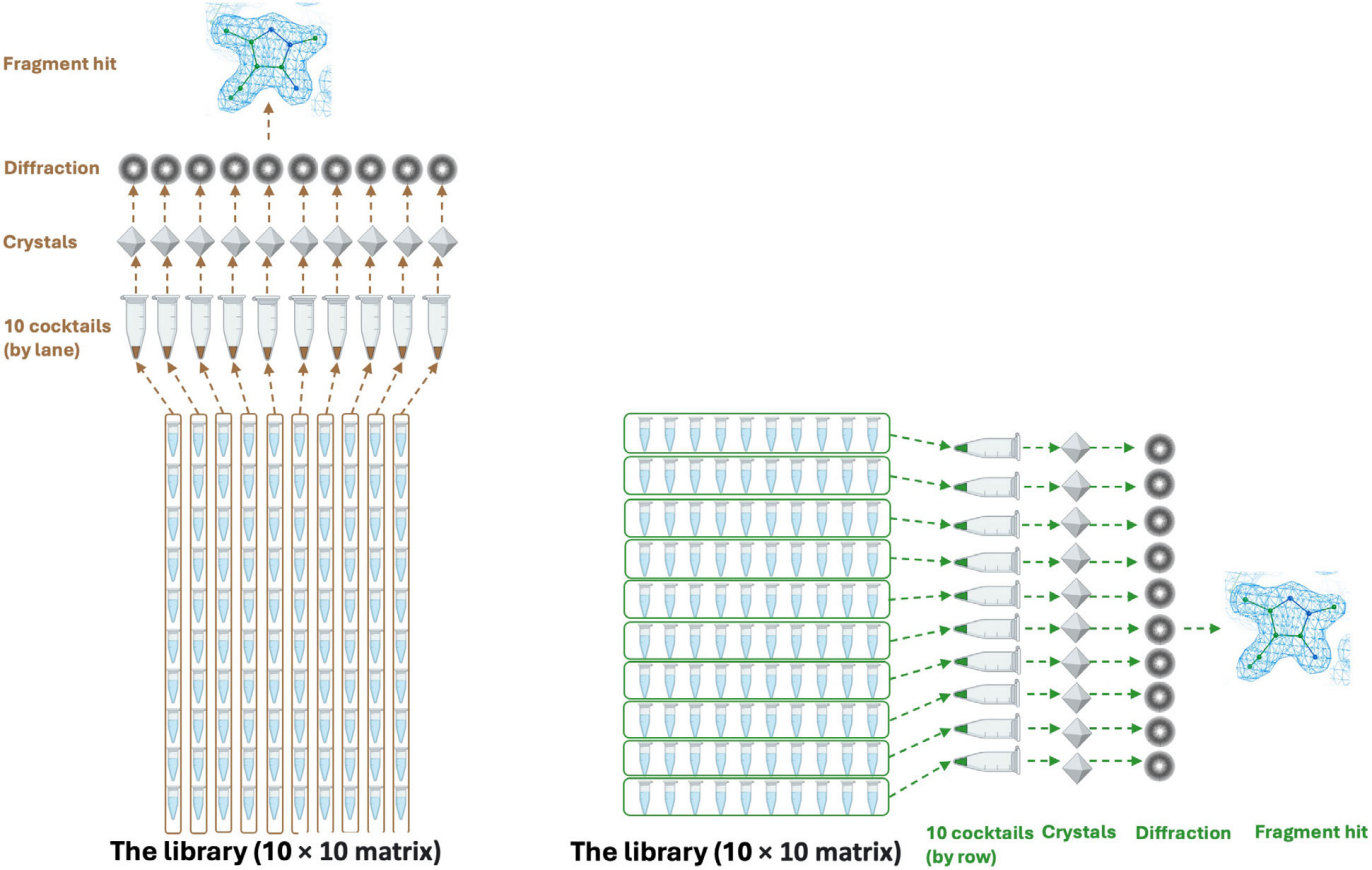
The construction of the fragment cocktail library. Fragment stocks were stored in Eppendorf tubes and assembled into a 10 × 10 matrix. The cocktailing was carried out by lanes (left panel) and rows (right panel), resulting in 20 different cocktails. These cocktails were applied to crystals by soaking, and protein structures were solved by X‐ray diffraction. The figure was generated by BioRender.

We next formatted LoCoFrag100 for cocktail soaking to enhance screening throughput. Fragments were arranged in a 10 × 10 matrix to minimize Tanimoto similarity in the 20 cocktails made from the rows and columns (Fig. 2). These cocktails were dried by a centrifugal vacuum concentrator and the resulting pellets of fragments were dissolved by the mother liquor of crystals, with the aim to reach approximately 140 mm of each fragment. No cryoprotectant (e.g., glycerol) was added into those cocktails. The cocktails (Table S2) were soaked with protein crystals to reach a maximum final concentration of approximately 70 mm if there was no precipitation. Based on this method, each screening campaign only cost approximately £2 of compounds, and every fragment is screened twice in the 20 independent soaking experiments. After soaking with cocktails, crystals were flash‐frozen in liquid nitrogen, and structures of the proteins discussed in the next section were solved by X‐ray crystallography. Fragments that only bound to crystal solvent channels were not deemed to be hits in this study.

### Fragment hits suggest new avenues to inhibit UDP‐GlcNAc pyrophosphorylase

We used UDP‐GlcNAc pyrophosphorylase (UAP1, EC 2.7.7.23) of *A. fumigatus* (*Af*UAP1) to investigate the potential uses of LoCoFrag100. This enzyme has very limited numbers of reported inhibitors [39, 52], with no published research on exploration of *Af*UAP1 PROTACs. Prior to this, we had carried out extensive fragment screening (≥ 1000 fragments) for this enzyme by conventional biophysical approaches, without resulting in any *Af*UAP1‐fragment complex structures. UAP1 is essential for the cell wall integrity of *A. fumigatus* as it is involved in the biosynthesis of UDP‐GlcNAc, the precursor of fungal cell wall chitin [36]. The structure of *A. fumigatus* UAP1 (*Af*UAP1) is a monomer consisting of an N‐terminal domain, a central domain, and a C‐terminal domain [36]. In the active site, *Af*UAP1 converts GlcNAc‐1P into UDP‐GlcNAc via S_N_2 substitution [39]. The enzymatic catalysis of UAP1 involves a conformational change [39, 53], and currently no information suggests which conformations are more ligandable. Here, we focussed on crystals of the *Af*UAP1‐GlcNAc‐1P complex (PDB 6TN3) [39] that diffract to 2.28 Å, with an asymmetric unit consisting of two conformationally distinct chains (apo‐like and GlcNAc‐1P‐bound).

**Fig. 3:**
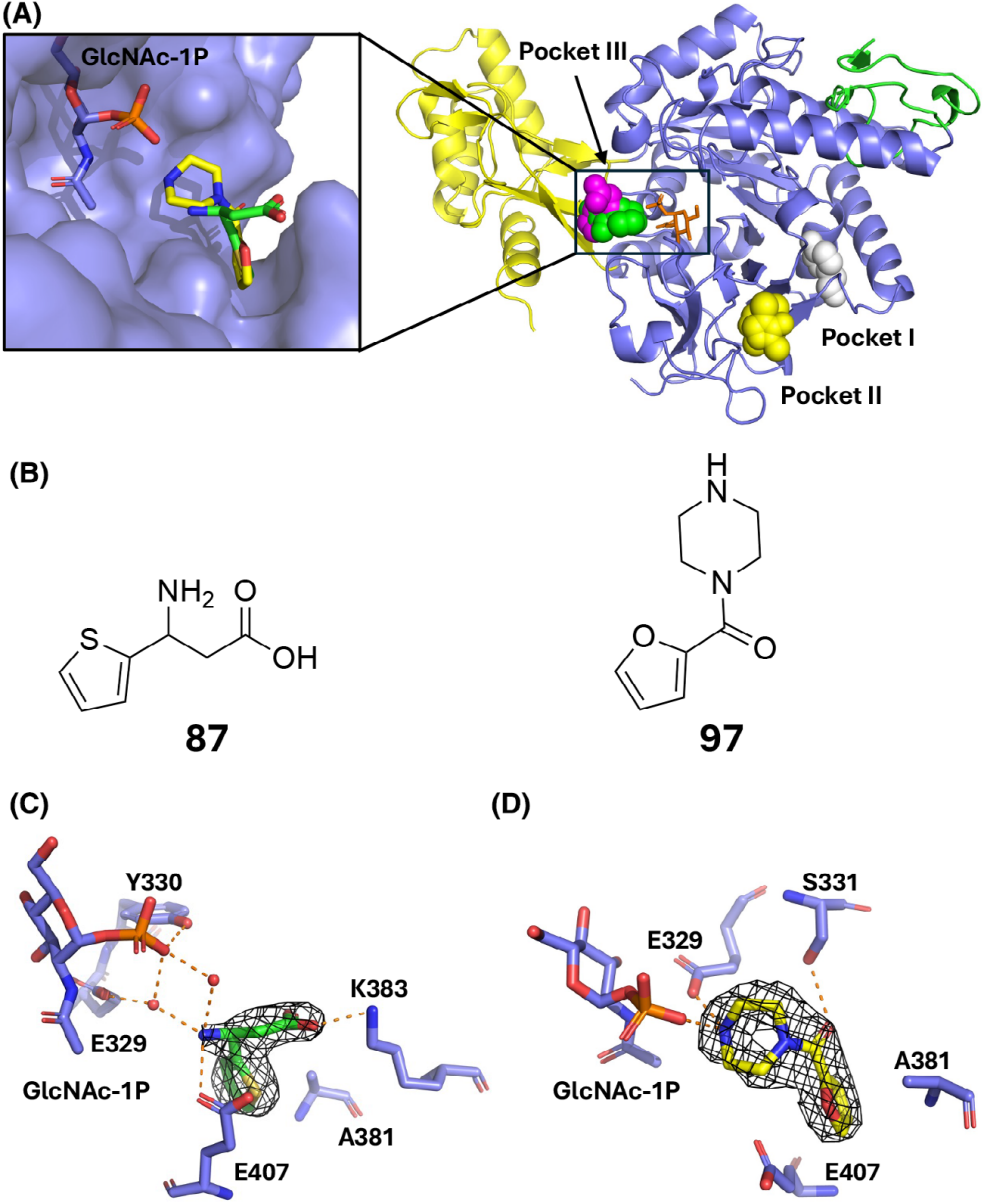
Crystal structures of *Af*UAP1 in complex with fragments. (A) Fragments bound to *Af*UAP1. The protein is shown as ribbon. The yellow ribbon represents the N‐terminal domain, blue represents the central domain, and green represents the C‐terminal domain. Spheres represent fragments (placed by superposition) bound to *Af*UAP1 in pockets (**I**, **II** and **III);** each colour indicates a different fragment. Orange sticks represent GlcNAc‐1P in the active site. The zoomed‐in panel shows the surface representation of pocket **III**. The protein is shown as blue surface. Yellow sticks represent fragment **97**, and green sticks represent fragment **87**. Fragments are overlayed in pocket **III** by superposition. (B) Structures of fragments (**87** and **97**) bound to *Af*UAP1. (C) Interactions between fragment **87** and *Af*UAP1 in pocket **III**. The fragment is shown in green. Black mesh indicates F_o_‐F_c_ map (contoured at 2.5σ) before inclusion of the ligand. Orange dashed lines represent hydrogen bonds. Red sphere represents a water molecule. The protein is shown in blue. (D) Interactions between fragment **97** and *Af*UAP1 in pocket **III**. The colour scheme is the same as that of (C) with the fragment shown in yellow. Black mesh represents F_o_‐F_c_ map (contoured at 2.5σ) before inclusion of the fragment.

From the 20 LoCoFrag100 cocktails, diffraction experiments identified four fragments (Fig. S2) from six crystals diffracting to approximately 1.6 Å (Table S3), equivalent to a hit rate of 4%. This hit rate is higher than that (0.1–0.2%) obtained from our previous fragment screening experiments [54, 55]. These fragment hits bind to three distinct pockets (**I**, **II** and **III**) on the *Af*UAP1 protein (Fig. 3A). Pockets **I** and **II** are on the central domain of *Af*UAP1 (Fig. 3A). Two fragments, **17** (found from Cocktails 8 and 14) and **121** (found from Cocktails 6 and 18), are bound to Pockets **I** and **II**, respectively (Figs 3A and S2). These two pockets are unique to fungal UAP1 enzymes and are not conserved in the human orthologue of UAP1 (hUAP1 or AGX1, Fig. S3). Although pockets **I** and **II** are far from the active site, they may provide possible starting points for the development of PROTACs with selectivity over hUAP1.

**Fig. 4:**
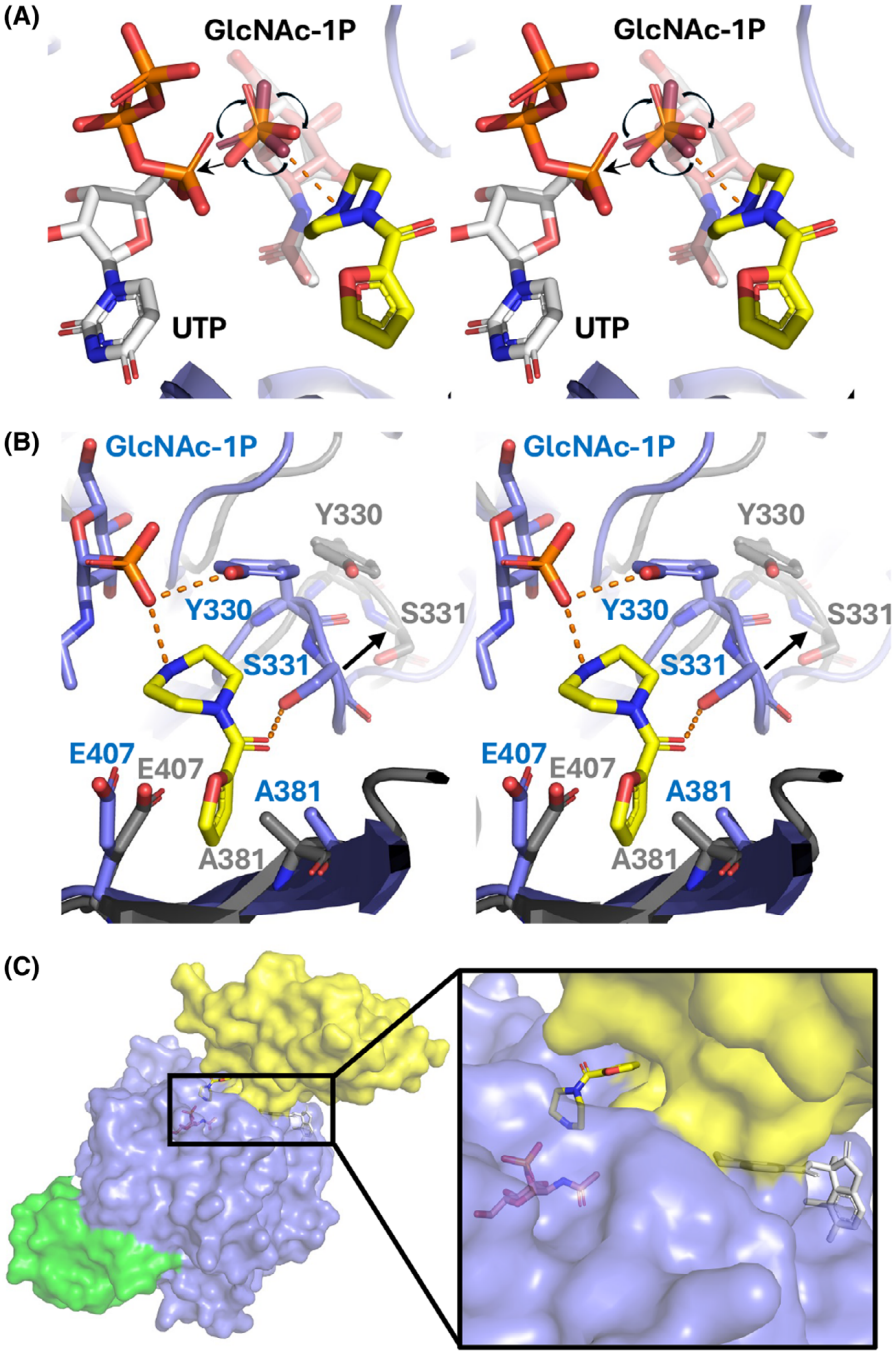
Structural comparison of fragment‐bound *Af*UAP1 with other proteins. (A) Blue ribbon represents the protein of the *Af*UAP1‐**97** complex, yellow sticks indicate fragment **97**. GlcNAc‐1P, in the *Af*UAP1‐**97** complex, is shown as orange sticks. Another GlcNAc‐1P molecule, from the native *Af*UAP1 protein (PDB 6TN3, no fragment soaked) [39], is placed by superposition and shown as white sticks. The UTP molecule (white sticks) is placed by superimposing the structure of *Af*UAP1‐UTP complex (PDB 6G9W) [39] onto that of the *Af*UAP1‐**97** complex. The straight arrow indicates the direction of nucleophilic attack. Dark red represents oxygen atoms on the phosphate group of GlcNAc‐1P in the *Af*UAP1‐**97** complex. The orange dashed line represents a hydrogen bond. Curved arrows indicate rotation of the phosphate group. The figure is shown in stereoscopic view. (B) Superposition of the *Af*UAP1‐**97** complex structure (blue) onto that of the apo *Af*UAP1 (grey), another chain in the same crystal of the *Af*UAP1‐**97** complex. Yellow sticks represent fragment **97**. The arrow indicates conformational change. Dashed lines represent hydrogen bonds. The figure is shown in stereoscopic view. (C) The surface represents the *Af*UAP1 protein in complex with **97** (yellow sticks). The yellow surface indicates the N‐terminal domain, blue indicates the central domain, and green represents the C‐terminal domain. Orange sticks represent GlcNAc‐1P in the *Af*UAP1‐**97** complex. White sticks represent the allosteric inhibitor of *Tb*UAP1 (PDB 4BQH) [52]. The allosteric inhibitor is placed by superposition of the *Tb*UAP1 structure onto that of *Af*UAP1.

Two other fragments (**87** and **97**, Fig. 3B) bind to a 158 Å^3^ pocket (Pocket **III**) that is 5 Å away from the active site (Fig. 3A). The thiophene ring of fragment **87** (found from Cocktails 4 and 16) stacks with the side chain carboxyl group of E407 and also interacts with the side chain of A381 via a CH‐*π* interaction (Fig. 3C). The carboxyl group of Fragment **87** coordinates the side chain amine of K383 via a salt bridge (Fig. 3C). The primary amine of Fragment **87** also interacts with E407 through a salt bridge (Fig. 3C). In addition, Fragment **87** also uses water‐mediated hydrogen bonds to interact with E329 and the GlcNAc‐1P substrate (Fig. 3C), and the *Af*UAP1‐**87** complex structures were solved from two (cocktail 4 and cocktail 16) of the 20 cocktails (Table 2). In the same pocket, the furan ring of rFagment **97** (found only from cocktail 18) mimics the thiophene of Fragment **87** (Fig. 3D). The carbonyl group of Fragment **97** forms a hydrogen bond with S331 (Fig. 3D), and the secondary amine of piperazine coordinates E329 and GlcNAc‐1P via a salt bridge (Fig. 3D). This salt bridge changes the orientation of the phosphate group towards this fragment (Fig. 4A). According to the catalytic mechanism of UAP1, the oxygen of this phosphate group participates in the nucleophilic attack that initiates S_N_2 substitution [39, 53], altering the phosphate conformation is likely to perturb the nucleophilic attack and in turn inhibit the catalytic process. As Pocket **III** is 5 Å away from the oxygen attacking α‐phosphate of UTP, it may be possible to perturb this nucleophilic attack with derivatives of Fragment **97**.

As mentioned previously, there are two protein chains (apo‐like and GlcNAc‐1P‐bound) in the asymmetric unit. Electron density maps of fragments only appear in the GlcNAc‐1P‐bound chain. We superimposed structures of *Af*UAP1‐fragment complexes onto the apo‐like *Af*UAP1 structure to understand why no fragment electron density is visible in the apo‐like chain. In the apo form of *Af*UAP1, the loop (residue 330 to 333) undergoes a 7 Å shift away from the active site compared to the substrate‐bound conformation (Fig. 4B). This movement disrupts the hydrogen bond between S331 and Fragment **97** (Figs 3D and 4B), perhaps reducing the binding affinity of this fragment. Furthermore, since the secondary amine of Fragment **97** coordinates the phosphate group of GlcNAc‐1P through a salt bridge (Fig. 3D), the absence of GlcNAc‐1P removes this salt bridge, reducing the binding affinity of Fragment **97**. As such, it is possible that the binding of Fragment **97** requires the presence of GlcNAc‐1P in the active site. Fragment **87** indirectly coordinates the GlcNAc‐1P via a water‐mediated hydrogen bond (Fig. 3C), and the presence of GlcNAc‐1P may also be essential for the binding of this fragment. Taken together, the presence of GlcNAc‐1P in the active site may contribute to the fragment binding, and a molecule that would represent the fusion of these fragments (**87** and **97**) to the GlcNAc‐1P scaffold may stabilize the substrate‐bound conformation and in turn prevent the binding of natural substrates.

The location of Pocket **III**, where Fragments **87** and **97** bind, is situated in the interface between the N terminus and central domains (Figs 3A and 4C), opposite to the binding pocket of a reported allosteric inhibitor of UAP1 [52] (Fig. 4C). The inhibitory mechanism of this allosteric inhibitor is to lock the apo‐form of UAP1 by binding to the same domain interface (N‐terminal and central domains), although on the other side of Pocket **III**. As such, Fragments **87** and **97** suggest another potential avenue to inhibit UAP1 via this domain interface (N terminus and central domains).

Unlike Pockets **I** and **II**, Pocket **III** is conserved in the monomeric form of hUAP1 (AGX1 and AGX2) (Fig. S3A). One feature to note is that AGX1, an isoform of hUAP1, can form biological dimers in solution [56]. Pocket **III** is in the dimer interface of AGX1 (Fig. S4). As such, the second monomer is likely to clash with fragments bound to the counterpart of Pocket **III** in AGX1 (Fig. S4). The dimerization of AGX1 suggests a possible strategy to preferentially inhibit *Af*UAP1. Taken together, two fragments binding adjacent to the natural ligand GlcNAc‐1P have been identified from the fragment cocktail library. These fragments, together with the GlcNAc‐1P scaffold, provide new avenues to explore inhibitors of *Af*UAP1.

### Fragment hits of phosphoglucose isomerase identified from LoCoFrag100

**Fig. 5:**
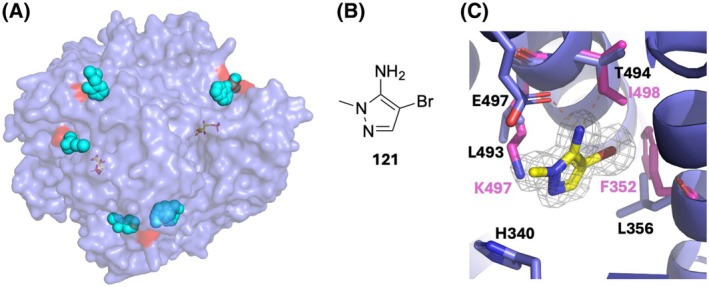
Crystal structures of *Ca*PGI in complex with fragment 121. (A) The structure of *Ca*PGI (purple surface) in complex with different fragments (spheres placed by superposition). Cyan spheres represent fragments on the protein surface. Red surface indicates residues that are not conserved in *Hs*PGI. Yellow sticks represent 5‐phosphoarabinonic acid in the active site. (B) The structure of fragment **121**. (C) Interactions between fragment **121** (yellow sticks) and *Ca*PGI (blue). Magenta sticks represent *Hs*PGI (PDB 1IRI) superimposed onto *Ca*PGI. Orange dashed lines represent hydrogen bonds. Grey mesh represents F_o_‐F_c_ map (contoured at 2.5σ) before inclusion of the fragment.

To further explore the general applicability of LoCoFrag100, we next applied this library to phosphoglucose isomerase (PGI, EC 5.3.1.9) of *A. fumigatus*, an enzyme that is involved in the biosynthesis of sugar nucleotides, precursors of the fungal cell wall [37]. To date, PGI inhibitors are mainly restricted to open‐chain pseudo‐substrates. *A. fumigatus* PGI (*Af*PGI) is a dimer in solution, and crystals that diffract to 1.6 Å have been reported [37]. However, 15 *Af*PGI crystals did not diffract after being soaked with fragment cocktails, possibly as a result of fragment‐induced conformational changes. As an alternative, we used the orthologue (*Ca*PGI) of *Af*PGI in *C. albicans*, for which no crystals/structures have as yet been reported. We crystallized *Ca*PGI (diffracted to 2.1 Å) and solved the structure by molecular replacement, using the structure of *Af*PGI as the search model [37]. Similar to *Af*PGI (Cα RMSD 0.3), the structure of *Ca*PGI is predicted as a dimer (buried area 6118 Å^2^) in solution by PISA [57], with each monomer harbouring two domains (PDB 9FZT). LoCoFrag100 was applied to crystals of *Ca*PGI in complex with a reported inhibitor (5‐phosphoarabinonic acid) that mimics the reaction intermediate [58]. As such, fragments binding adjacent to this inhibitor could be potential starting points for the synthesis of larger, more specific/potent molecules. After being soaked with cocktails (Table S3), five fragments (only found once) were identified on surface pockets of *Ca*PGI (Figs. 5A, S5), with an overall hit rate of 6% (Table 2). Although these five fragments do not suggest immediate avenues to develop inhibitors, their binding sites are close to non‐conserved residues (Fig. 5A), suggesting that these fragments may be suitable starting points of PROTACs with selectivity over human PGI. One fragment (**121**, Fig. 5B) appears from Cocktails 6 and 18 (Table 2) and is buried into a previously unknown pocket (with nonconserved residues) in the interior of the *Ca*PGI structure (Fig. 5C). Although these fragments do not immediately reveal clear avenues to explore the synthesis of PGI inhibitors, these data demonstrate that *Ca*PGI is ligandable with the LoCoFrag100 library.

### Identification of an active site fragment for dTDP‐4‐dehydrorhamnose reductase

**Fig. 6:**
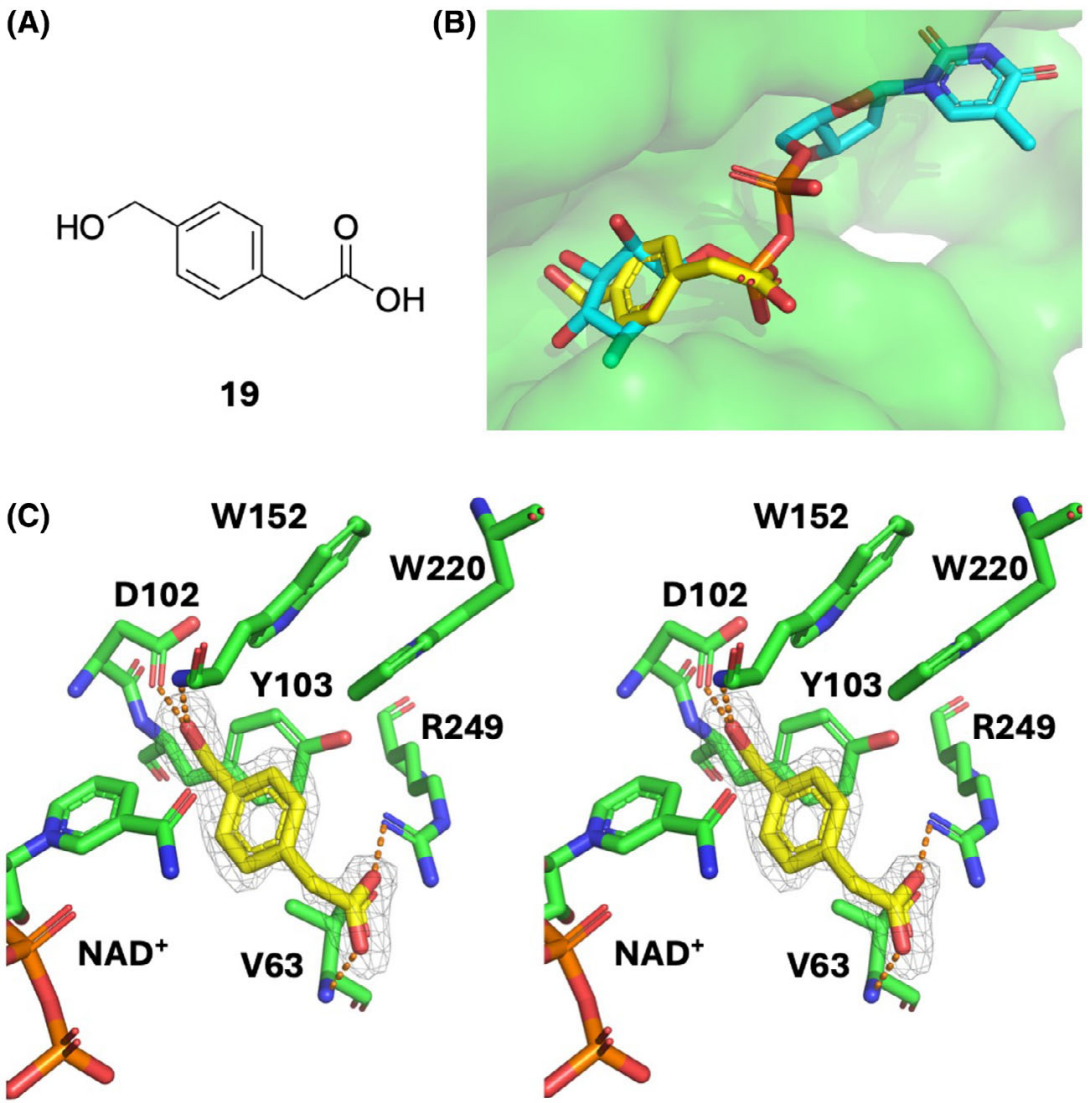
Crystal structures of GacA in complex with fragment 19. (A) Chemical structures of fragments **19**. (B) Surface representation of the GacA‐fragment complex structure. GacA is shown as a green surface. Fragment **19** is shown as yellow sticks. The substrate (dTDP‐rhamnose, cyan sticks) is placed by superimposing the GacA structure onto that of the RmlD‐substrate complex (PDB 1KC3). (C) Interactions between GacA (green) and fragment **19** (yellow sticks). Orange dash lines indicate hydrogen bonds. Black mesh indicates F_o_‐F_c_ map (contoured at 2.5σ) before inclusion of the fragment. The figure is shown in stereoscopic view.

Having demonstrated that LoCoFrag100 is functional in two fungal proteins, we next sought to apply this to enzymes involved in bacterial cell wall synthesis that are considered antibacterial targets [59]. Group A *Streptococcus* (GAS) is a pathogenic bacterium leading to > 0.5 million deaths annually [60]. The cell wall of GAS is made up of peptidoglycan, lipoteichoic acid, hyaluronan and Group A Carbohydrate (GAC) [61]. Rhamnose sugars form the GAC backbone, which is further decorated with GlcNAc side chains and glycerol‐phosphate [62]. The precursor of the rhamnose residue is dTDP‐rhamnose, which is converted from dTDP‐6‐deoxy‐L‐mannose with the cofactor NADP^+^ by dTDP‐4‐dehydrorhamnose reductase (EC 1.1.1.133, RmlD or GacA) [38, 61]. Being an essential enzyme of GAS, GacA serves as a new target against this bacterial pathogen [38]. The GacA protein from Gram‐positive bacteria forms a monomer binding the substrate and cofactor in a long cleft [38]. Previously we have carried out fragment screening (1000 fragments) for GacA using the conventional biophysical approach but did not obtain GacA‐fragment complex structures [63]. Here LoCoFrag100 was applied to well‐characterized [38] crystals of GacA that routinely diffract to 1.6 Å, identifying Fragment **19** (only found once) (Figs 6A and S6) as a hit (Table S3). By superimposing the GacA‐fragment **19** complex structure onto the GacA‐dTDP‐rhamnose complex [64], we found that this fragment binds in the active site of GacA (Fig. 6B). The benzene ring of Fragment **19** mimics the rhamnose ring of the substrate (Fig. 6B). The hydroxyl group of the fragment forms hydrogen bonds with D102 and W152 (Fig. 6C). The carboxyl group of the fragment mimics the diphosphate group of the substrate (Fig. 6B), forming a salt bridge with R249 and a hydrogen bond with V63 (Fig. 6C). As such, although the final hit rate of targeting GacA is only 1%, Fragment **19** was identified as a potential starting scaffold to develop inhibitors of GacA in GAS.

## Concluding remarks

While fragment‐based approaches have been widely used in the early‐stage drug discovery as well as fundamental research, the identification of fragments remains a challenging task. Although sophisticated facilities (e.g., XChem) improved the chances of success [27, 29], their application is limited by practical issues related to accessibility and cost. Here we designed LoCoFrag100, a cocktail library allowing fast fragment crystallographic screening in any standard structural biology laboratory. The hit rate of LoCoFrag100 ranges from 1 to 6% (Table 2) for the three targets explored in this study, which compares favourably to other reported libraries (Table 3). Although MiniFrags [51] shows a significantly higher average hit rate of 44% for five targets (kinases, protein–protein interaction targets and a metalloenzyme), this is mainly due to the fact that MiniFrags consists of ultra small molecules, which have a higher chance to bind effectively to proteins (Table 3). Moreover, the entire LoCoFrag100 library is covered twice by 20 crystal soaking experiments, and this number is much lower than that (at least 80 crystals) required by commercial crystallographic libraries [50, 51]. As such, LoCoFrag100 is arguably more efficient (with reduced time and resources for growing crystals) than current commercial crystallographic libraries whilst also providing sufficient solid compound material for repeat and potential follow‐up activities cost‐effectively. The increased efficiency suggests that LoCoFrag100 could be used to quickly assess the ligandability of a certain target, and that ligandability justifies the feasibility of subsequent more extensive crystallographic screening in sophisticated facilities (e.g., XChem), which may require application for facility slots with peer‐reviewed decisions. For instance, we recently used Fragments **87** and **97** to apply for XChem beamline time for *Af*UAP1, and the application has successfully passed peer review (Proposal No. LB41355). A criticism of LoCoFrag100 is that this library is small, and therefore, the coverage of chemical space is limited. This limitation does introduce the potential to miss relevant chemotypes, but precedented literature examples [15, 65, 66] demonstrate that sub‐1000 (even sub‐500) member fragment sets can yield hits that can develop into tool compounds and higher‐quality leads. As such, we suggest mitigation of this limitation by emphasizing the need for high‐information structural readouts and, where possible, rapid follow‐up screening and/or chemistry for elaboration. Another limitation of the fragment ‘cocktailing approach’ is the potential for unambiguous assignment of ligand identity from the initial screen as a result of complex or overlapping fragment binding modes. Cocktailing only structurally dissimilar compounds, as carried out in this study, likely helped mitigate this limitation. A practical drawback of this fragment cocktail approach is an increased likelihood of crystal cracking leading to loss of diffraction, as became apparent for *Af*PGI. Moreover, since LoCoFrag100 is designed as a crystallographic screening library, binding affinity of fragment hits cannot be directly obtained from complex structures. However, this is a broad disadvantage of X‐ray crystallography, and this disadvantage can be overcome through determination of fragment binding affinity in follow‐up experiments. Furthermore, weak‐binding fragments (millimolar affinity) can still be derived into high‐affinity inhibitors (nanomolar affinity) based on the structural information of ligand binding sites [4, 67]. This demonstrates that the lack of apparent binding affinity does not automatically mean fragment hits lack merit. Nevertheless, we have demonstrated that positive results can be obtained from three structurally distinct proteins (*Ca*PGI, GacA and *Af*UAP1), suggesting that the library is universally functional and can be applied to other targets in the future.

**Table 3:** Summary of the crystallographic screening of the fragment cocktail library.

	*Ca*PGI	GacA	*Af*UAP1
Cocktail 1	No fragment identified	No fragment identified	No fragment identified
Cocktail 2	No fragment identified	Crystal destroyed	Crystal did not diffract
Cocktail 3	No fragment identified	No fragment identified	No fragment identified
Cocktail 4	No fragment identified	No fragment identified	**87**
Cocktail 5	No fragment identified	**19**	Low resolution (2.9 Å)
Cocktail 6	**121**	Crystal destroyed	**121**
Cocktail 7	No fragment identified	Crystal destroyed	Low resolution (2.7 Å)
Cocktail 8	No fragment identified	No fragment identified	**17**
Cocktail 9	Crystal did not diffract	No fragment identified	Crystal did not diffract
Cocktail 10	No fragment identified	Crystal destroyed	No fragment identified
Cocktail 11	No fragment identified	Crystal destroyed	Data cannot process
Cocktail 12	**66**	No fragment identified	No fragment identified
Cocktail 13	**217, 88**	Data cannot process	No fragment identified
Cocktail 14	No fragment identified	No fragment identified	**17**
Cocktail 15	No fragment identified	Crystal destroyed	No fragment identified
Cocktail 16	**74**	No fragment identified	**87**
Cocktail 17	No fragment identified	Crystal destroyed	Low resolution (2.8 Å)
Cocktail 18	**121**	No fragment identified	**97, 121**
Cocktail 19	**136**	Crystal destroyed	Low resolution (3.5 Å)
Cocktail 20	No fragment identified	No fragment identified	Crystal did not diffract
Hit rate (%)	6	1	4

$$\mathrm{Hit}\;\text{rate}=\frac{\text{Number of identifed fragment}}{\text{Number of total fragments}}$$

In summary, we have designed a new fragment cocktail library (LoCoFrag100) for crystallographic screening. LoCoFrag100 is functional among structurally distinct proteins and with improved features: low cost, common chemical vendor, only 20 crystals required. Therefore, LoCoFrag100 serves as a tool to quickly evaluate the protein ligandability (assuming access to appropriate facilities), which forms the basis for further focussed or additional crystallographic screening at specific facilities (e.g., XChem (Diamond Light Source, UK)). Moreover, our work also serves as the first study in dissecting the ligandability of the three cell wall targets (UAP1, PGI and GacA), revealing new avenues to target microbial cell walls.

## Author contributions

MS, KY and DMFvA were involved in conceptualization and methodology; KY, MS, OR, ATF, HD and DMFvA. were involved in investigation; KY, MS, HD and DMFvA. were involved in formal analysis; ATF and HD were involved in resources; KY, HD and DMFvA. were involved in writing – original draft; MS, OR and ATF. were involved in writing – review and editing; DMFvA was involved in project administration and funding acquisition.

## Supporting information


**Fig. S1.** Comparison of LoCoFrag100 and other libraries.
**Fig. S2.** Electron density maps of fragments bound to *Af*UAP1.
**Fig. S3.** Superposition of the structure of *Af*UAP1 (blue ribbon) onto that of hUAP1 (magenta ribbon, PDB 1JV1).
**Fig. S4.** The biological dimer of hUAP1 (AGX1, PDB 1JV1).
**Fig. S5.** Electron density maps of fragments bound to *Ca*PGI.
**Fig. S6.** Electron density maps of fragments bound to GacA.

**Table S1.** Comparison of LoCoFrag100 with commercial libraries (Jena and MiniFrag).

**Table S2.** General properties of fragments.

**Table S3.** X‐ray diffraction summary.

**Data S1.** Similarity and dissimilarity matrix of fragments.

**Data S2.** Practical guidance of LoCoFrag100.

**Data S3.** The script to generate 10 × 10 matrix.

## Data Availability

The library information, including the matrix and fragments of cocktails, is included in the Supporting Information. The fragment concentration of each stock is also provided in the Supporting Information. The price of each fragment is shown as part of the Supporting Information. Crystallographic datasets (Figs 3, 4, 5, 6 and S2–S6) are deposited in the RCSB Protein Data Bank (PDB), and PDB entry codes of datasets are included in Table S3.
