# Supplementary material for: Cell wall target fragment discovery using a low‐cost, minimal fragment library
Source: FEBS Lett. 2026 Jan 14;600(11):1638–54. doi: 10.1002/1873-3468.70281 (PMC13244422; doi:10.1002/1873-3468.70281)
Supplement: Supplementary file 1 — Fig. S1. Comparison of LoCoFrag100 and other libraries. Fig. S2. Electron density maps of fragments bound to AfUAP1. Fig. S3. Superposition of the structure of AfUAP1 (blue ribbon) onto that of hUAP1 (magenta ribbon, PDB 1JV1). Fig. S4. The biological dimer of hUAP1 (AGX1, PDB 1JV1). Fig. S5. Electron density maps of fragments bound to CaPGI. Fig. S6. Electron density maps of fragments bound to GacA. [file FEB2-600-1638-s004.pdf]

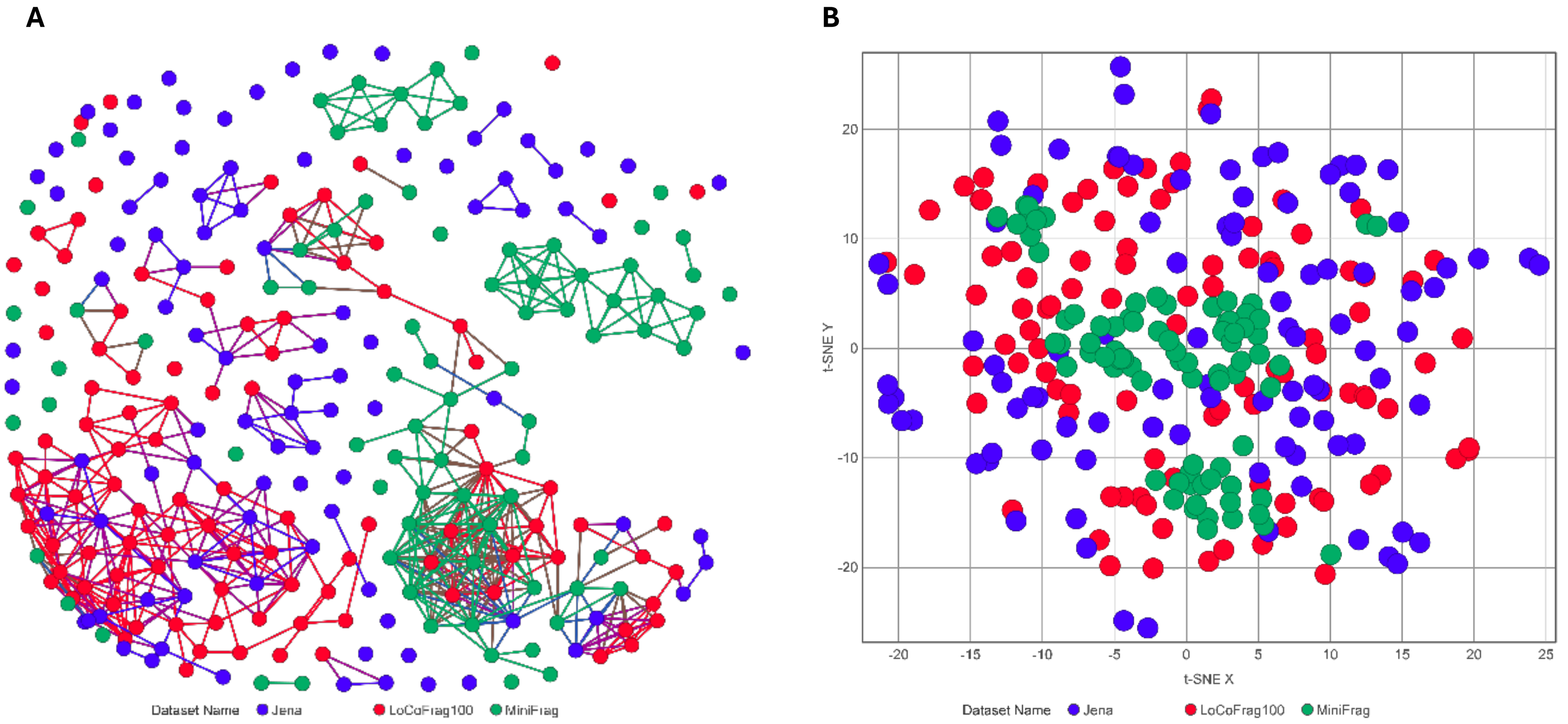

Figure S1. Comparison of LoCoFrag100 and other libraries. (A) Chemical similarity analysis ('Flexophore fingerprint') of libraries (LoCoFrag100, Jena and MiniFrag) using DataWarrior. Similarity relationships are represented by the physical distances between the graphical datapoints. (B) t-SNE (T-distributed Stochastic Neighbour Embedding) visualization of chemical similarity of libraries (LoCoFrag100, Jena and MiniFrag).

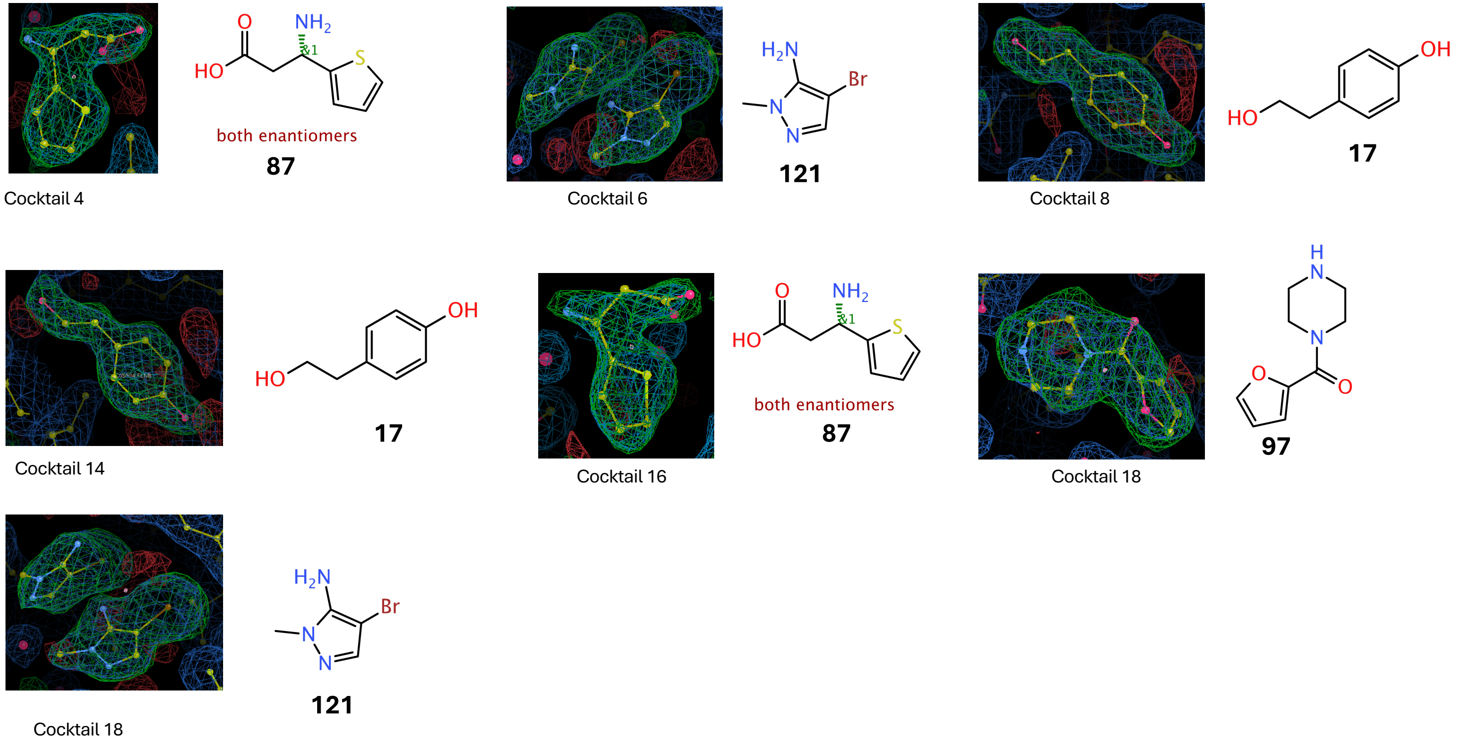

Figure S2. Electron density maps of fragments bound to *Af*UAP1. Blue mesh indicates 2Fo-Fc map contoured at 1σ. Green mesh represents Fo-Fc map (contoured at 2.5σ) before inclusion of ligands.

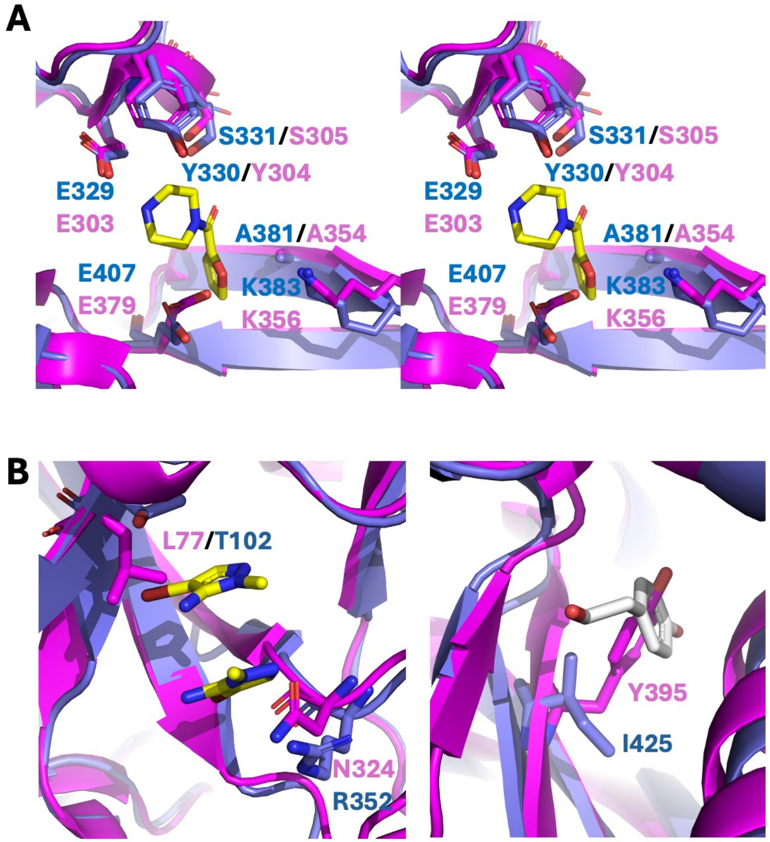

Figure S3. Superposition of the structure of *Af*UAP1 (blue ribbon) onto that of hUAP1 (magenta ribbon, PDB 1JV1). (A) Fragment **97** is shown as yellow sticks. The figure is shown as stereoscopic view. (B) Fragments **121** (in pocket **II**) and **17** (in pocket **I**) are shown in yellow and white, respectively.

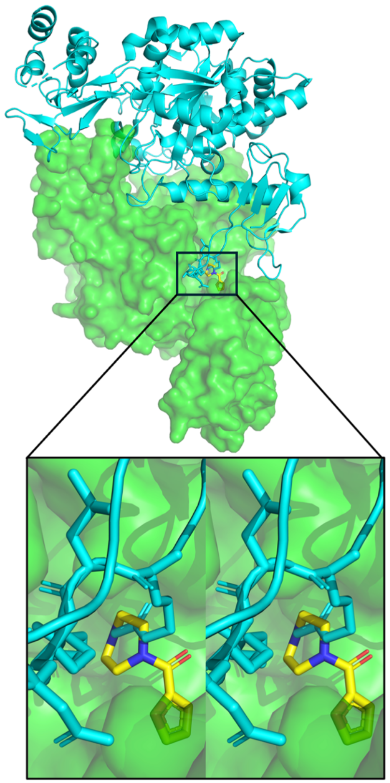

Figure S4. The biological dimer of hUAP1 (AGX1, PDB 1JV1). Monomers are shown in cyan and green. Fragment **97** (yellow sticks) is placed by superimposing the structure of *Af*UAP1-**97** complex onto that of the AGX1 monomer (green). The dimer interface is zoomed in and shown in stereoscopic view.

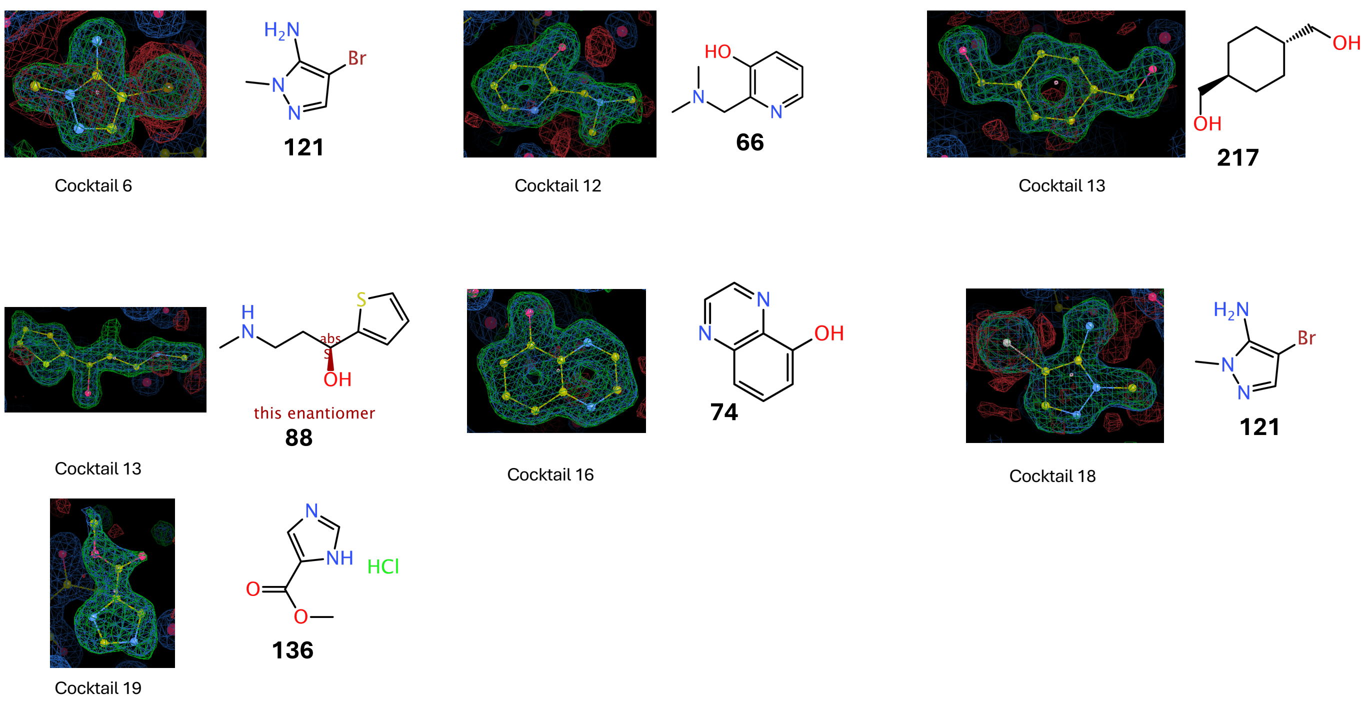

Figure S5 Electron density maps of fragments bound to *Ca*PGI. Blue mesh represents 2Fo-Fc map contoured at 1σ, and green mesh represents Fo-Fc map (contoured at 2.5σ) before inclusion of ligands.

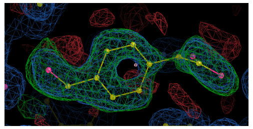

Cocktail 5

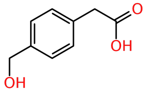

19

Figure S6. Electron density maps of fragments bound to GacA. Blue mesh represents 2Fo-Fc map contoured at 1σ. Green mesh indicates Fo-Fc map (contoured at 2.5σ) before inclusion of ligands.
