# Supplementary material for: Cell wall target fragment discovery using a low‐cost, minimal fragment library
Source: FEBS Lett. 2026 Jan 14;600(11):1638–54. doi: 10.1002/1873-3468.70281 (PMC13244422; doi:10.1002/1873-3468.70281)
Supplement: Supplementary file 6 — Data S2. Practical guidance of LoCoFrag100. [file FEB2-600-1638-s003.pdf]

## Practical guidance of LoCoFrag100

**Cocktailing by lanes** (columns) / **Cocktailing by rows** (rows)

| | Cocktail 1 | Cocktail 2 | Cocktail 3 | Cocktail 4 | Cocktail 5 | Cocktail 6 | Cocktail 7 | Cocktail 8 | Cocktail 9 | Cocktail 10 |
|---|---|---|---|---|---|---|---|---|---|---|
| Cocktail 11 | 139 | 40 | 27 | 128 | 19 | 180 | 171 | 219 | 145 | 93 |
| Cocktail 12 | 11 | 209 | 163 | 134 | 189 | 221 | 133 | 25 | 55 | 66 |
| Cocktail 13 | 208 | 88 | 18 | 54 | 131 | 23 | 190 | 153 | 118 | 217 |
| Cocktail 14 | 192 | 127 | 61 | 211 | 41 | 57 | 137 | 17 | 176 | 164 |
| Cocktail 15 | 24 | 116 | 72 | 167 | 187 | 210 | 150 | 79 | 222 | 32 |
| Cocktail 16 | 148 | 62 | 123 | 87 | 74 | 162 | 216 | 95 | 21 | 203 |
| Cocktail 17 | 64 | 215 | 197 | 5 | 165 | 184 | 85 | 126 | 37 | 113 |
| Cocktail 18 | 108 | 151 | 212 | 218 | 97 | 121 | 154 | 52 | 7 | 191 |
| Cocktail 19 | 185 | 90 | 188 | 16 | 224 | 42 | 80 | 146 | 136 | 220 |
| Cocktail 20 | 44 | 147 | 132 | 170 | 140 | 130 | 63 | 223 | 174 | 20 |

**10 x 10 matrix.** Each number represents a fragment

## Cocktail 1

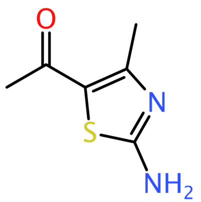
139

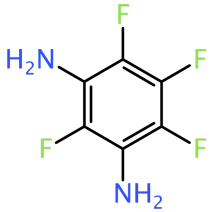
11

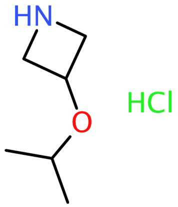
208

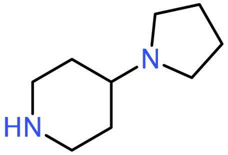
192

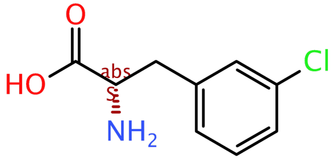
this enantiomer
24

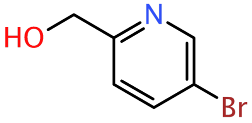
64

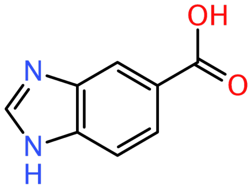
108

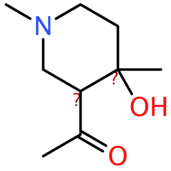
unknown chirality
185

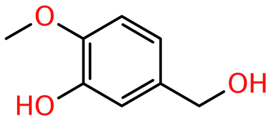
44

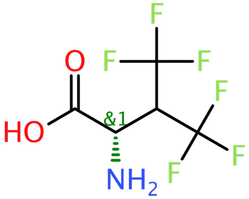
both enantiomers
148

## Cocktail 2

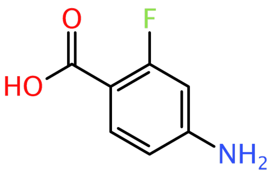

40

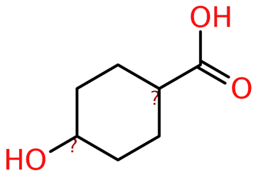

209

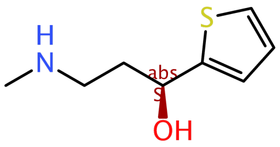

this enantiomer

88

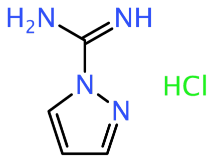

127

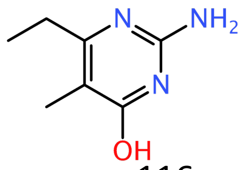

116

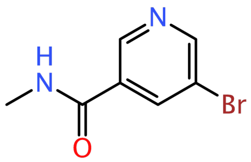

62

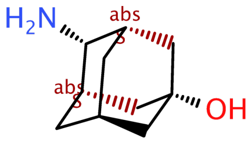

this enantiomer

215

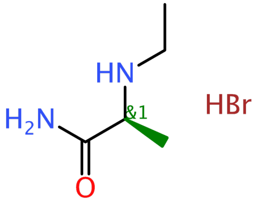

both enantiomers

151

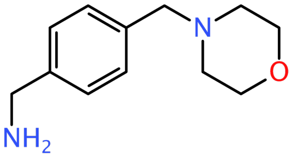

90

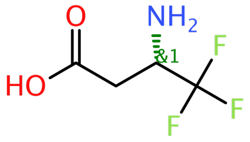

both enantiomers

147

**Cocktail 3**

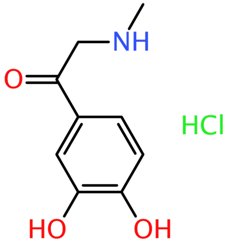

27

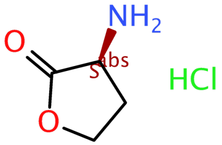

this enantiomer

163

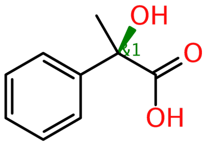

both enantiomers

18

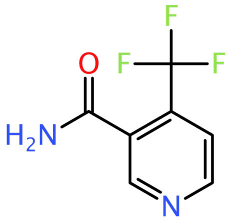

61

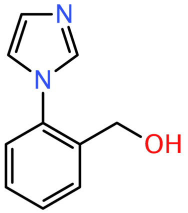

72

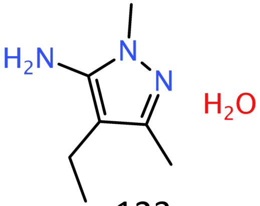

123

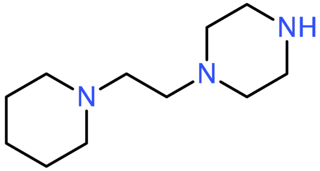

197

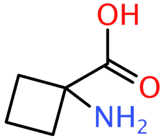

212

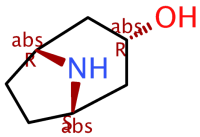

meso

188

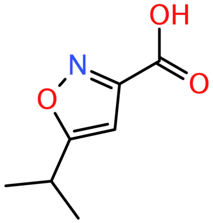

132

# Cocktail 4

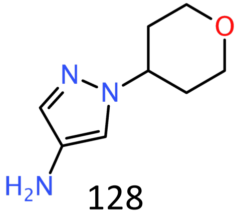

128

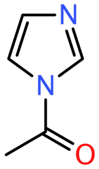

134

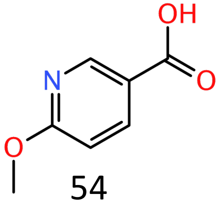

54

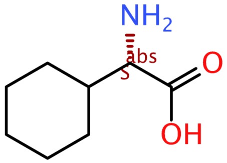

this enantiomer
211

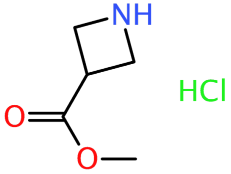

167

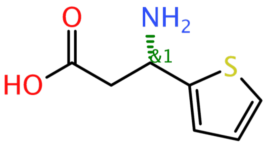

both enantiomers
87

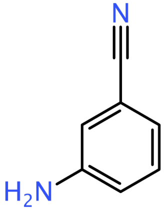

5

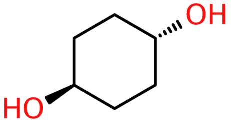

218

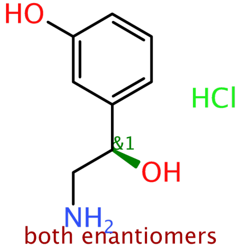

both enantiomers
16

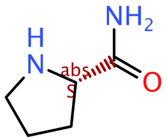

this enantiomer

170

## Cocktail 5

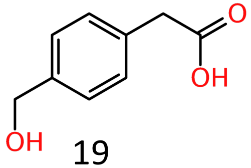

19

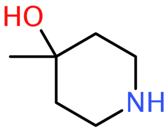

189

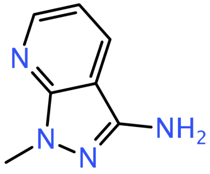

131

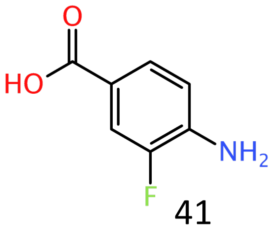

41

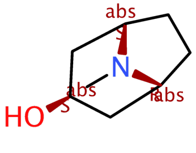

meso

187

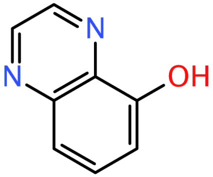

74

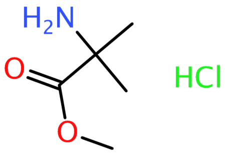

165

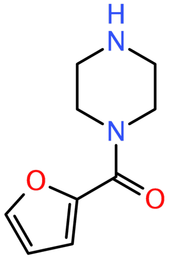

97

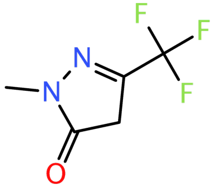

224

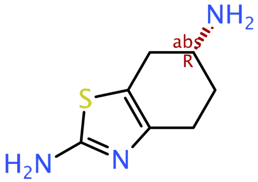

this enantiomer

140

# Cocktail 6

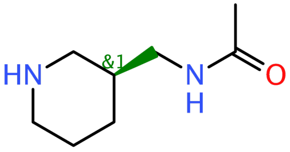

both enantiomers
180

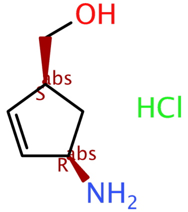

this enantiomer
221

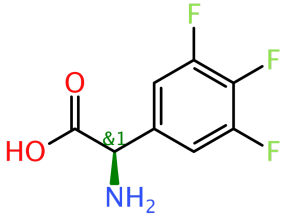

both enantiomers
23

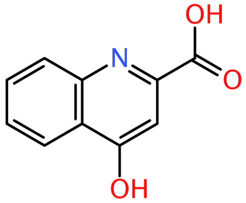

57

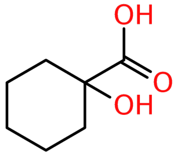

210

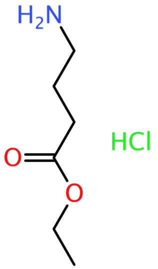

162

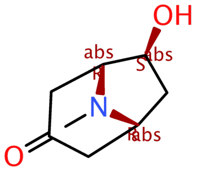

this enantiomer
184

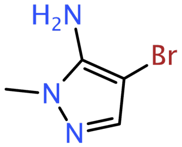

121

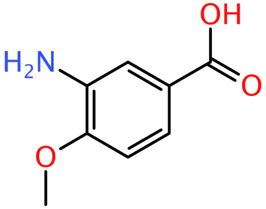

42

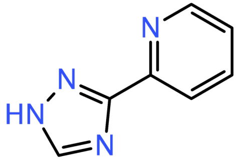

130

# Cocktail 7

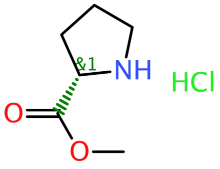

both enantiomers

171

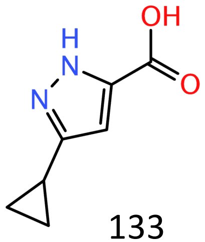

133

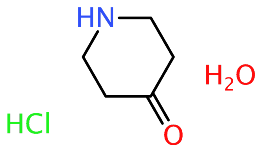

190

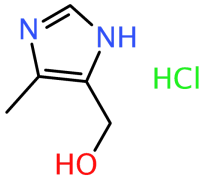

137

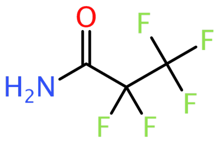

150

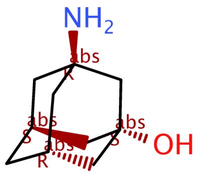

meso

216

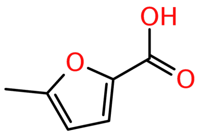

85

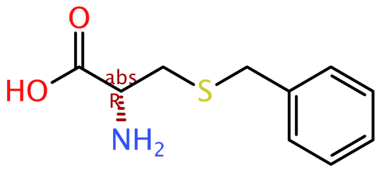

this enantiomer

154

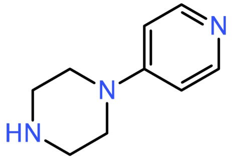

80

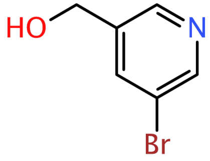

63

# Cocktail 8

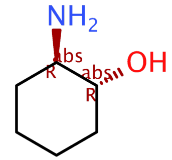


this enantiomer

219

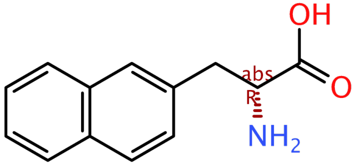


this enantiomer

25

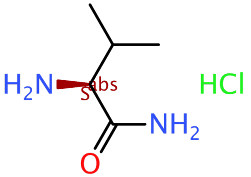


this enantiomer

153

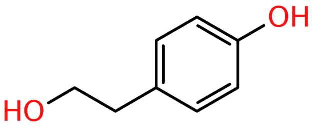


17

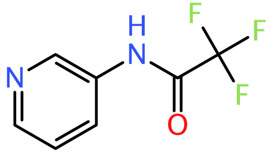


79

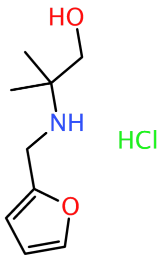


95

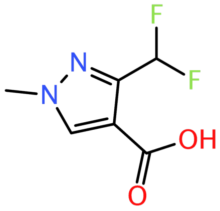


126

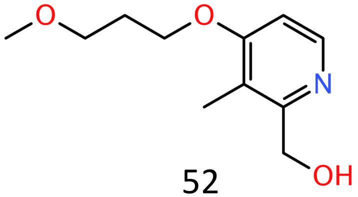


52

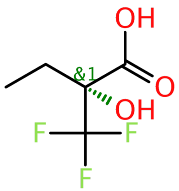


both enantiomers

146

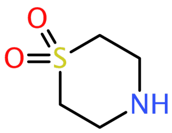


223

# Cocktail 9

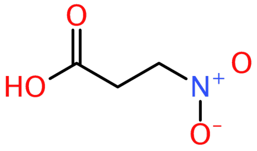

145

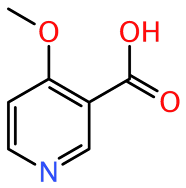

55

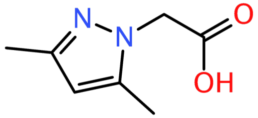

118

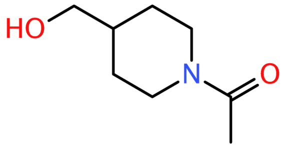

176

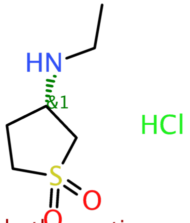

both enantiomers

222

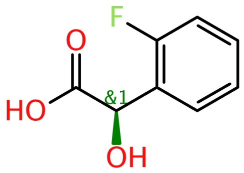

both enantiomers

21

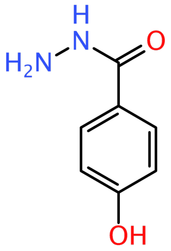

37

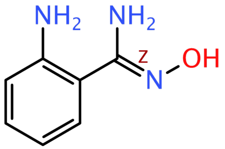

7

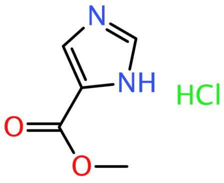

136

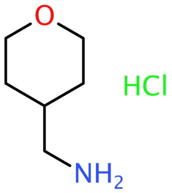

174

## Cocktail 10

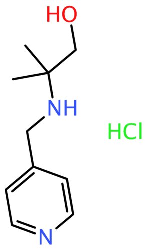


93

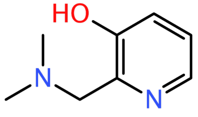


66

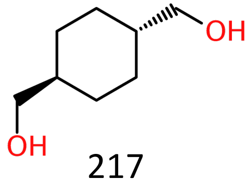


217

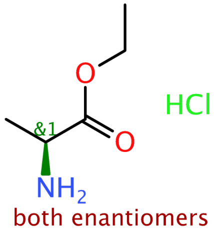


164

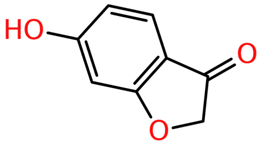


32

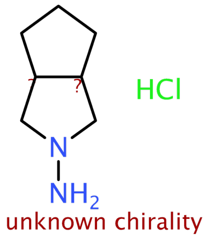


203

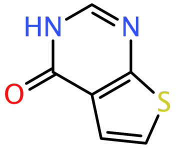


113

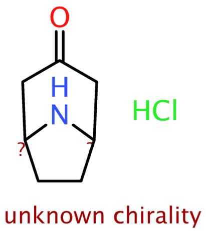


191

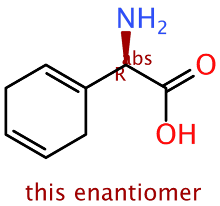


220

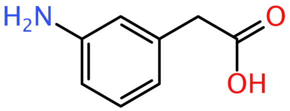


20

# Cocktail 11

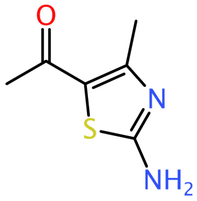

139

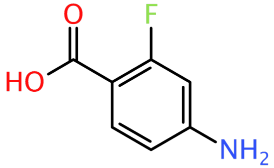

40

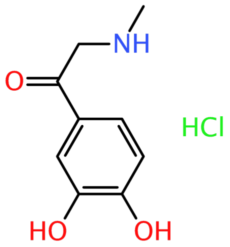

27

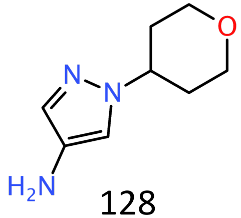

128

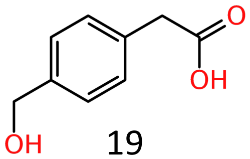

19

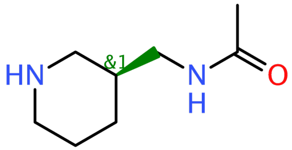

both enantiomers

180

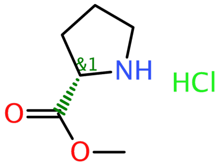

both enantiomers

171

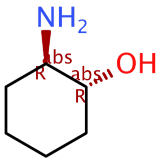

this enantiomer

219

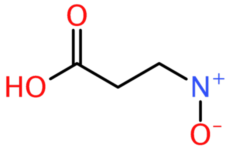

145

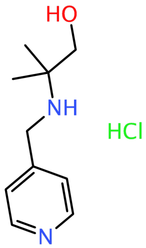

93

# Cocktail 12

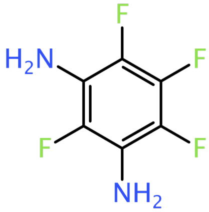
11

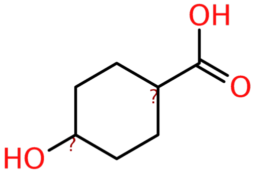
209

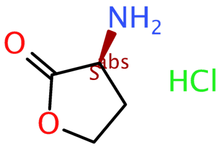
this enantiomer
163

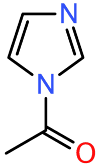
134

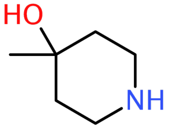
189

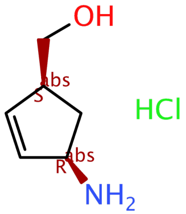
this enantiomer
221

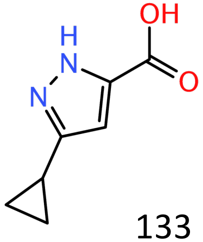
133

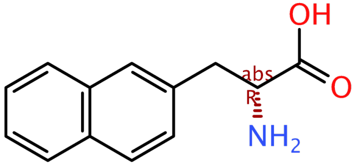
this enantiomer
25

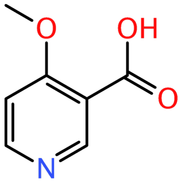
55

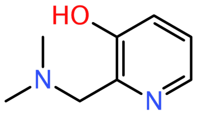
66

# Cocktail 13

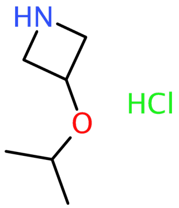

208

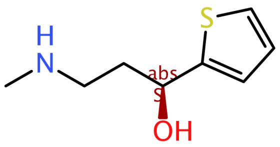

this enantiomer

88

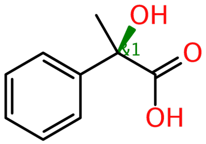

both enantiomers

18

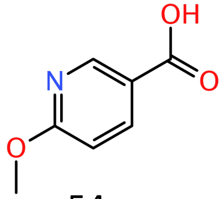

54

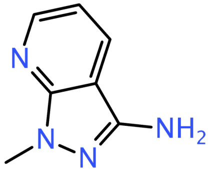

131

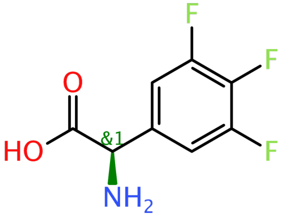

both enantiomers

23

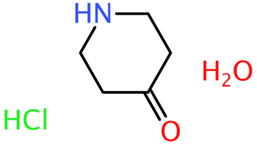

190

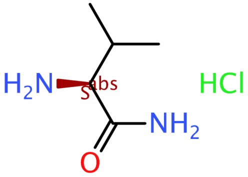

this enantiomer

153

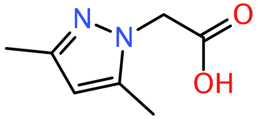

118

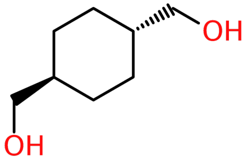

217

**Cocktail 14**

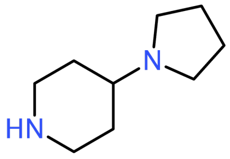

192

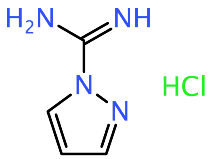

127

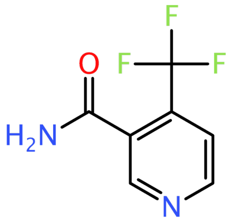

61

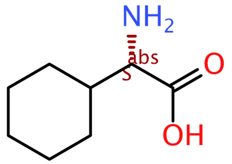

this enantiomer

211

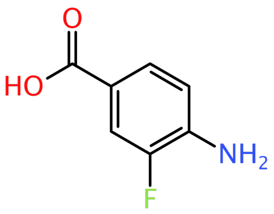

41

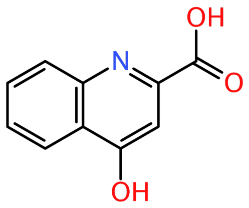

57

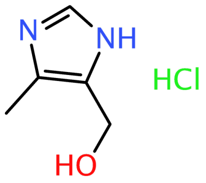

137

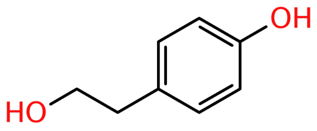

17

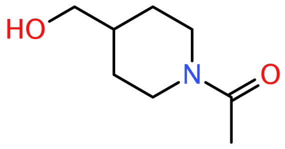

176

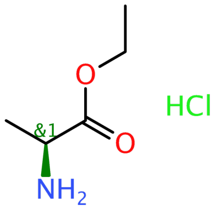

both enantiomers

164

# Cocktail 15

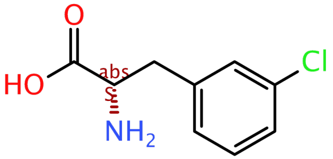

this enantiomer

24

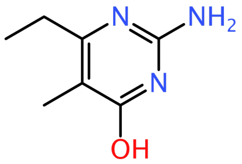

116

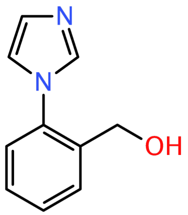

72

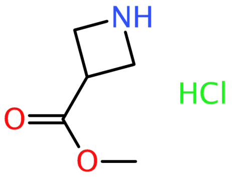

167

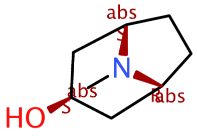

meso

187

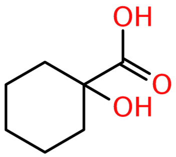

210

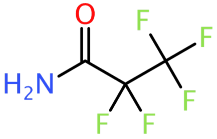

150

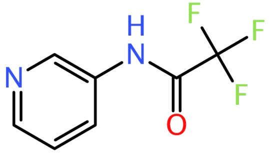

79

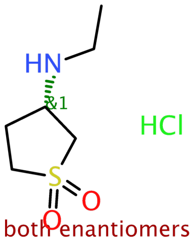

both enantiomers

222

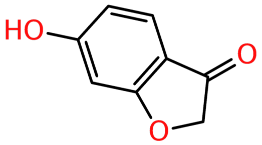

32

# Cocktail 16

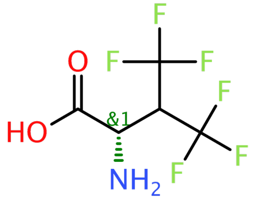

148

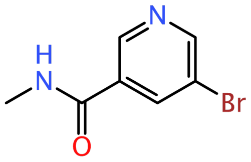
62

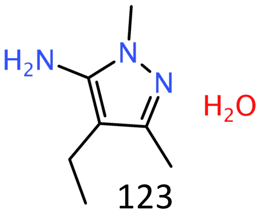
123

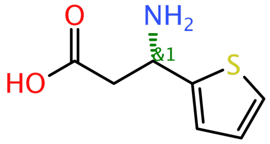

87

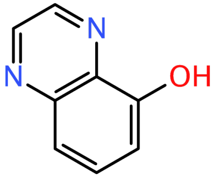
74

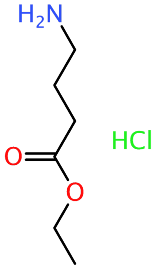
162

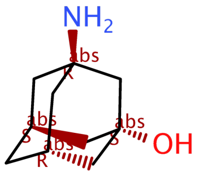

216

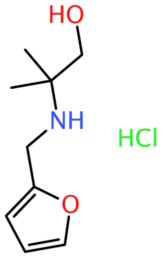
95

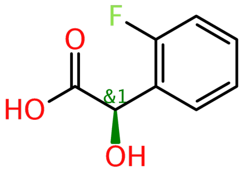

21

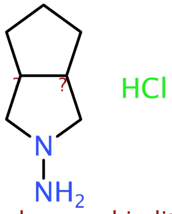

203

# Cocktail 17

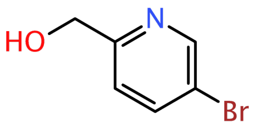
64

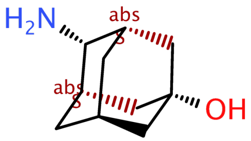
this enantiomer
215

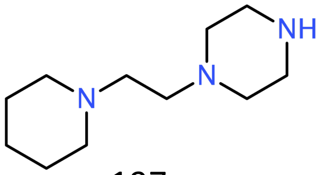
197

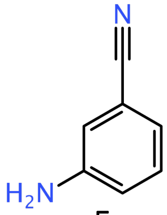
5

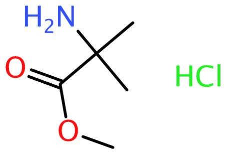
165

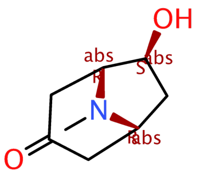
this enantiomer
184

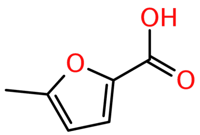
85

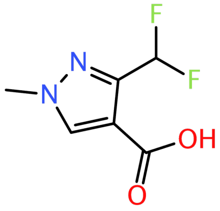
126

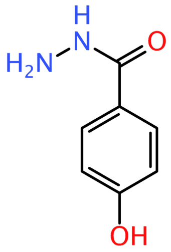
37

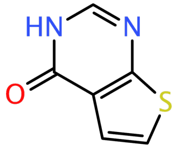
113

# Cocktail 18

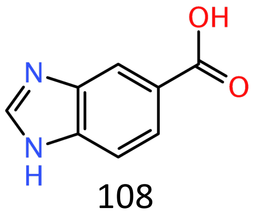

108

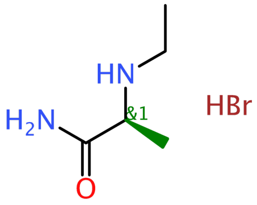


both enantiomers

151

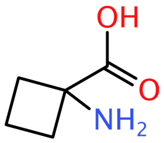

212

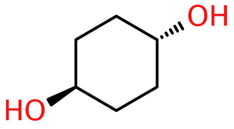

218

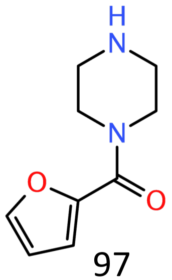

97

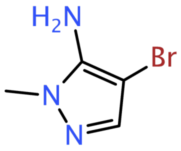

121

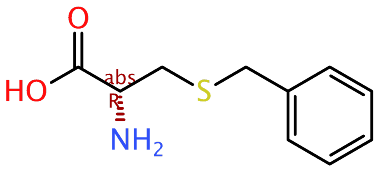

this enantiomer

154

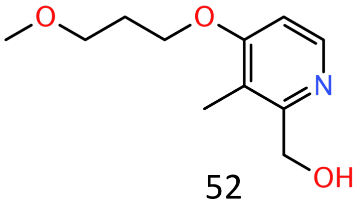

52

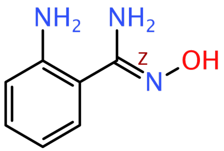

7

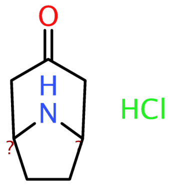


unknown chirality

191

# Cocktail 19

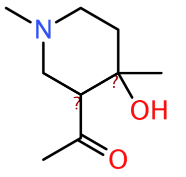

unknown chirality

185

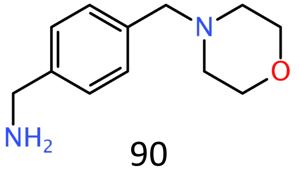

90

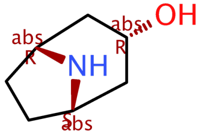

meso

188

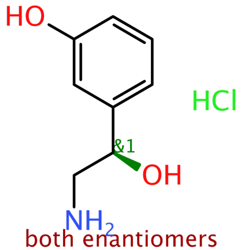

both enantiomers

16

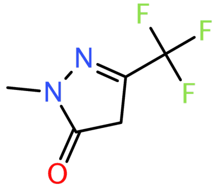

224

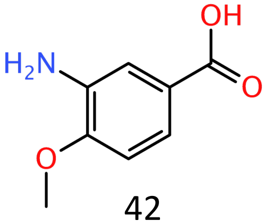

42

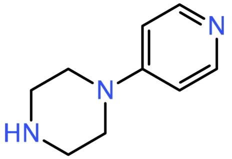

80

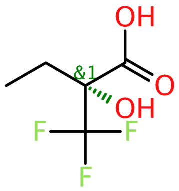

both enantiomers

146

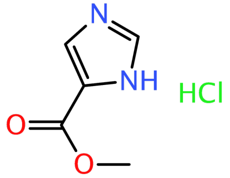

136

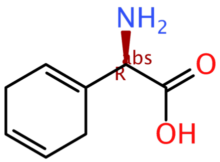

this enantiomer

220

# Cocktail 20

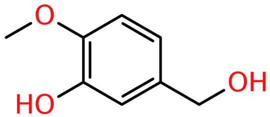

44

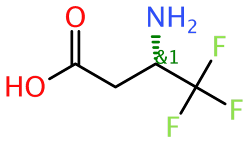

both enantiomers

147

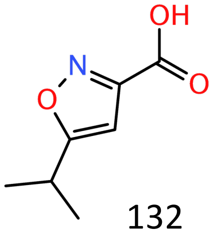

132

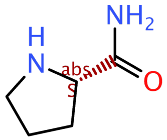

this enantiomer

170

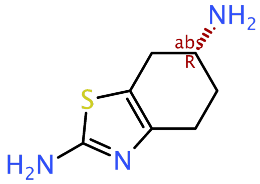

this enantiomer

140

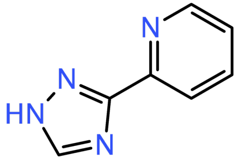

130

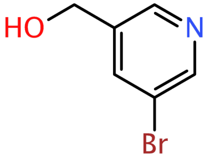

63

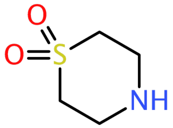

223

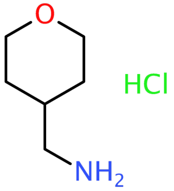

174

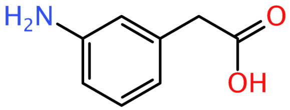

20
